# Brassinosteroids affect wood development and properties of *Fraxinus mandshurica*

**DOI:** 10.3389/fpls.2023.1167548

**Published:** 2023-07-17

**Authors:** Han Lu, Mingjun Chen, Meng Fu, Jialin Yan, Wenlong Su, Yaguang Zhan, Fansuo Zeng

**Affiliations:** ^1^ State Key Laboratory of Tree Genetics and Breeding, Northeast Forestry University, Harbin, China; ^2^ College of Life Science, Northeast Forestry University, Harbin, China

**Keywords:** *Fraxinus mandshurica*, hybridization, wood property variations, WGCNA, brassinosteroids

## Abstract

**Introduction:**

Xylem development plays a crucial role in wood formation in woody plants. In recent years, there has been growing attention towards the impact of brassinosteroids (BRs) on this xylem development. In the present study, we evaluated the dynamic variation of xylem development in Fraxinus mandshurica (female parent, M8) and a novel interspecific hybrid *F. mandshurica* × *Fraxinus sogdiana* (1601) from May to August 2020.

**Methods:**

We obtained RNA-Seq transcriptomes of three tissue types (xylem, phloem, and leaf) to identify the differences in xylem-differentially expressed genes (X-DEGs) and xylem-specifically expressed genes (X-SEGs) in M8 and 1601 variants. We then further evaluated these genes via weighted gene co-expression network analysis (WGCNA) alongside overexpressing FmCPD, a BR biosynthesis enzyme gene, in transient transgenic *F. mandshurica*.

**Results:**

Our results indicated that the xylem development cycle of 1601 was extended by 2 weeks compared to that of M8. In addition, during the later wood development stages (secondary wall thickening) of 1601, an increased cellulose content (14%) and a reduced lignin content (11%) was observed. Furthermore, vessel length and width increased by 67% and 37%, respectively, in 1601 compared with those of M8. A total of 4589 X-DEGs were identified, including enzymes related to phenylpropane metabolism, galactose metabolism, BR synthesis, and signal transduction pathways. WGCNA identified hub X-SEGs involved in cellulose synthesis and BR signaling in the 1601 wood formation–related module *(CESA8, COR1, C3H14,* and *C3H15)*; in contrast, genes involved in phenylpropane metabolism were significantly enriched in the M8 wood formation–related module (*CCoAOMT* and *CCR*). Moreover, overexpression of *FmCPD* in transient transgenic *F. mandshurica* affected the expression of genes associated with lignin and cellulose biosynthesis signal transduction. Finally, BR content was determined to be approximately 20% lower in the M8 xylem than in the 1601 xylem, and the exogenous application of BRs (24-epi brassinolide) significantly increased the number of xylem cell layers and altered the composition of the secondary cell walls in *F. mandshurica*.

**Discussion:**

Our findings suggest that BR biosynthesis and signaling play a critical role in the differing wood development and properties observed between M8 and 1601 *F. mandshurica*.

## 1 Introduction

*Fraxinus* spp. (ash species) are hardwood forest tree species widely distributed throughout Asia, America, and Europe (Wallander, 2013; Sollars et al., 2017). *Fraxinus* spp. wood is considered particularly valuable due to its hardness, beautiful texture, and color, making it suitable for fine woodwork, such as that in the manufacture of furniture, sports equipment, and tool handles (Panshin and De, 1970). In recent years, the infectious pathogen *Hymenoscyphus pseudoalbidus* and the emerald ash borer (*Agrilus planipennis*) have spread to most parts of Europe and North America, resulting in continuous destructive damage of ash trees (Herms and McCullough, 2014; George et al., 2022). Despite being seemingly unaffected by the aforementioned biological threats, *Fraxinus mandshurica* has encountered alternative problems such as excessive logging, slow growth, and climate change in Northeast China; this species has, consequently, been listed as an endangered plant under secondary protection (Fu and Jin, 1992). The increasing demand for *F. mandshurica* wood prompted our research into the interspecific hybridization of *Fraxinus* spp.

Producing hybrid species via crossbreeding is a common and effective strategy of forest breeding. This strategy produces plants that are considered superior to their parental counterparts in terms of yield, growth rate, vitality, and disease resistance (Al-Ahmad, 2018). Interspecific hybridization is one form of distant hybridization. By exchanging genetic information between different species within the same genus, high-quality traits can be integrated into cultivated seeds, thus enabling the breeding of different plant variants and the creation of germplasms (Alix et al., 2017). To cultivate new hardwood varieties with rapid growth and superior wood properties, we conducted hybridization experiments using *Fraxinus* spp. plants. In a previous study, the interspecific hybrid clones *Fraxinus americana* × *F. mandshurica* and *Fraxinus velutina* × *F. mandshurica* were determined to exhibit faster growth rates and greater stress resistance than their female parent, *F. mandshurica* (Zeng et al., 2014; Zeng et al., 2015; He et al., 2016). Recently, a novel interspecific hybrid variant clone of *F. mandshurica*, ‘Dongshui1601’, has been cultivated by hybridizing *F. mandshurica* with *Fraxinus sogdiana*. The male parent *F. sogdiana* (2-1) was collected from Xinjiang Province, China, and the female parent *F. mandshurica* (M8) was collected from a forest farm in Heilongjiang Province, China.

Wood is a natural, renewable, resource-rich, and non-polluting biomass material, which is also known as the secondary xylem and is produced by the vascular cambium of internal meristems. Overall, the most strikingly divergent characteristics between different wood types are their corresponding anatomical structures and chemical compositions, especially within the initial stages of latewood development. During this stage, tree height stops increasing, xylem tissue becomes denser, and wood properties become more stable (Mitchell, 1958). Hormones, signal transduction pathways, transcriptional regulation, and post-transcriptional regulatory factors have been established as the main factors controlling the formation of secondary xylem (wood) (Jacobs, 1952; Yamamoto et al., 1997; Aspeborg et al., 2005; Ye and Zhong, 2015; Wang et al., 2019). Plant hormones, such as brassinosteroids (BRs), contribute to wood formation control; further, these hormones play a vital role in the domestication and hybridization of different species (Sun et al., 2018; Gupta et al., 2021). Over the last decade, there has been increasing focus on how BRs affect xylem biosynthesis in woody plants (Jin et al., 2014; Shen et al., 2018; Jiang et al., 2021). In addition, genes encoding enzymes involved in secondary cell wall (SCW) cellulose, hemicellulose, and lignin production have been discovered, most of which have been functionally characterized in *Populus* and *Eucalyptus* spp. (Shi et al., 2010; Zhong and Ye, 2014; Ye and Zhong, 2015; Kim et al., 2019). High-throughput sequencing, bioinformatics, and network analysis strategies have provided improved methods for analyzing the intricate genetic linkages underlying the physiological processes of wood formation among different tree species (Langfelder and Horvath, 2008; Chang et al., 2019). Ultimately, this provides a technical method by which we can evaluate the in-depth mechanism of wood formation.

*F. mandshurica* is an important broadleaved hardwood tree found in Northeast China. It possesses a ring-porous wood structure and exhibits distinct differences in development at the latewood stage. In the present study, we observed differences in the wood formation of *F. mandshurica* × *F. sogdiana* (1601) and *F. mandshurica* (M8) clones over time. Specifically, we focused on physiological traits related to growth rate and wood composition combined with joint analysis using RNA sequencing (RNA-seq). Our primary objectives were to distinguish the variations in wood properties and molecular regulation mechanisms of *F. mandshurica* (M8) and *F. mandshurica* × *F. sogdiana* (1601) and to provide a foundation for the molecular breeding and hybridization of *F. mandshurica*.

## 2 Materials and methods

### 2.1 Plant growth conditions and materials

In April 2020, flowering male branches of *F. sogdiana* (2-1) were collected from Xinjiang Province, China. These branches were then cultivated in water, and their pollen was collected in test tubes, which were stored at 4°C. Hybridization was performed in May 2020 using *F. mandshurica* (M8) and *F. sogdiana* as the female and male parents, respectively, in Dailing, Heilongjiang Province (47.03°N, 129.03°E), China. Interspecific hybrid progenies and intraspecific open-pollinated plants from parental *F. mandshurica* and *F. sogdiana* were planted in the Maoershan experimental forest farm of Northeast Forestry University under natural conditions (45.14°N, 127.55°E) in a randomized complete block design; each region contained 10 plants with six replicates. A novel *F. mandshurica* hybrid progeny variant ‘1601’ (authorized by the national new plant variety in China: 20190287), which exhibits superior growth traits, especially in the xylem development cycle and leaf size, was cultivated.

For BR treatments, 288 μl of stock solution, containing 10 mg/ml of 24-epiBrassinolide (BL; PhytoTech Laboratories, Lenexa, KS, USA) in ethanol, was added to 100 ml of 1/2MS culture medium (PhytoTech Laboratories) to give an approximate concentration of 10 μM of BL. Open-pollinated mature M8 seeds were collected in mid-September (2020) from Dailing, Heilongjiang Province, China. The sterilized seeds were transferred to 100 ml of 1/2MS culture medium for 2 weeks and then transferred to 1/2MS with 10 μM of BL for 3 weeks. The sterilized seeds were grown in a greenhouse under the following conditions: 14-h-light/10-h-dark, 25°C ± 1°C, and 70% ± 10% humidity.

### 2.2 Histochemical staining and scanning electron microscopy analysis of stem samples

A cross-section was taken from the 3-year-old plant stems 1.3 m above ground level using a trephor (Rossi et al., 2006). Then, cells were fixed in FAA buffer (formaldehyde:glacial acetic acid:50% ethanol, 1:1:18). Samples were taken counterclockwise at 2–4-cm intervals. The samples were removed and placed into a 1.5-ml centrifuge tube containing 70% alcohol solution. The astra blue/safranin dye method was used for histochemical staining to identify lignified and unlignified tissues, with astra blue dyeing the unlignified area blue and safranin dyeing the lignified area red (Dox et al., 2020). After being embedded in paraffin, the stems were cross-sectioned using an ultrathin semiautomatic microtome (FINESSE 325; Thermo Fisher Scientific, Waltham, MA, USA) and stained with 0.15% (w/v) astra blue and 0.04% (w/v) safranin mixed dye for 10 min. The cells were then washed twice with deionized water and observed under an optical microscope (Stemi-508; Zeiss, Oberkochen, Germany). For free-hand sectioned samples, the fast green/safranin dye method was used. Meanwhile, the wood cells were separated after treatment with an acetic acid/hydrogen peroxide (1:1, v/v) solution at 83°C for 8 h. The separated wood cells were then stained with safranin (1% in water); the width or length of the fiber cells and vessels, xylem relative area, and SCW thickness were then measured under a microscope (BX53, Olympus, Tokyo, Japan) using ImageJ software (version 1.8.0) (Long et al., 2013). Each sample consisted of three biological replicates (n = 3).

### 2.3 Chemical analysis of secondary cell wall components

Xylem samples were used for chemical analysis of SCW components on 15 July 2020. Total lignin content was measured using the classical Klason lignin method (Bhagia et al., 2016). Cellulose content was measured using concentrated sulfuric acid hydrolysis (Camacho et al., 1996). Hemicellulose content was determined by combining hydrochloric acid hydrolysis with 3,5-dinitrosalicylic acid (Foster et al., 2010).

### 2.4 RNA-seq library and differentially expressed gene analysis

In July 2020, the bark and phloem of the plant stems were peeled off 1.3 m above ground level; the xylem and phloem were collected from each tree group by scraping with a single-head razor. Meanwhile, the third functional leaf was collected as the leaf tissue sample. All samples were immediately frozen in liquid nitrogen. Each sample (M8X, M8 xylem; M8CP, M8 phloem; M8L, M8 leaf; 1601X, 1601 xylem; 1601CP, 1601 phloem; and 1601L, 1601 leaf) contained three biological replicates (n = 3). An improved cetyltrimethylammonium bromide method was used to extract the total RNA (Jaakola et al., 2001). RNA was precipitated using ethanol and dissolved in RNase-free water. Then, 1 µg of the pooled RNA was used for cDNA synthesis and library construction. Next, high-quality RNA was used for library preparation and paired-end sequencing (2 × 100 bp) using the DNPSEQ platform, according to the manufacturer’s instructions. Raw reads were filtered using SOAPnuke (version 1.4.0) and aligned against the *F. mandshurica* × *F. sogdiana* (1601) and *F. mandshurica* (M8) transcriptomes using Bowtie 2 (version 2.2.5). Gene counts were obtained from the resulting BAM files using RSEM (version 1.2.3), and gene expression was normalized to the expected number of fragments per kilobase of transcript sequence per million base pairs sequenced (FPKM). Differential analyses were conducted using the R package edgeR (Robinson et al., 2010). The Beijing Genomics Institute Co., Ltd. (Beijing, China) constructed and sequenced the RNA library. The clean reads were mapped to the *Fraxinus excelsior* reference genome (http://www.ashgenome.org/transcriptomes; Annotation Version 4, TGAC v2; assembly BATG-0.5) (Sollars et al., 2017) using the software Spliced Transcripts Alignment to a Reference (version 2.5.0). DESeq2 v1.4.5 was used for differential gene expression analysis between *F. mandshurica* (M8) and *F. mandshurica* × *F. sogdiana* (1601) using three biological replicates (n = 3) and a model based on negative binomial distribution. A p-value was assigned to each gene and adjusted using Benjamini and Hochberg’s method for controlling false discovery rate. Genes with a log2 fold change >1 or <−1 and a q-value ≤ 0.05 in each pairwise comparison were identified as xylem differentially expressed genes (X-DEGs).

### 2.5 Gene Ontology and Kyoto Encyclopedia of Genes and Genomes analysis of DEGs

The DEGs were identified between three biological replicates (n = 3) of *F. mandshurica* (M8) and *F. mandshurica* × *F. sogdiana* (1601) using the GOseq R packages (Young et al., 2010). The Gene Ontology (GO) distribution of these DEGs was then obtained at three levels: biological process (BP), molecular function (MF), and cellular component (CC). KOBAS software was used for the Kyoto Encyclopedia of Genes and Genomes (KEGG) pathway enrichment analysis to identify pathways that were significantly enriched in DEGs. The “phyper” package for the R computing platform was used to calculate the p-values, as previously described (Pan et al., 2016).

### 2.6 Weighted gene co-expression network analysis of xylem specifically expressed genes

DEGs were identified among the three tissues (xylem, phloem, and leaf) of M8 and 1601 plants to screen for genes that are specifically expressed in the xylem. The xylem specifically expressed genes (X-SEGs) in *F. mandshurica* (M8) and *F. mandshurica* × *F. sogdiana* (1601) were filtered by employing the following criteria: tissue DEGs with a log2 fold change (X vs. CP or L) ≥ 1 (q-value ≤ 0.05) were assigned as upregulated genes in the xylem compared to those in the phloem and leaves; DEGs with a log2 fold change (X vs. P or L) ≤ −1 (q-value ≤ 0.05) were assigned as downregulated genes in the xylem compared to those in the two other tissue types. X-SEGs are represented by the intersection between X vs. CP and X vs. L. Weighted gene co-expression network analysis (WGCNA) was performed using the R package WGCNA (version 4.0.3) (Langfelder and Horvath. 2008) to construct the co-expression networks for these genes. Genes with a low abundance (FPKM ≥ 1, FPKM value < 0.5) were filtered to eliminate noise. Co-expression modules were then built using the automatic network construction function “block-wise modules” with default settings, except for the following alterations: soft power = 30, merge cut height value = 0.25, minimum module size = 30, and minimum height for merging modules = 0.3131. Based on these criteria, weighted co-expression clusters were obtained, which were visualized as heatmaps according to the correlation coefficients of the modules. The color depth was used to represent the correlation between the module and the degree of vernalization. GO and KEGG pathway analyses were conducted for each module per species, and the modules that were enriched in genes associated with wood formation were identified.

### 2.7 Analysis of hub genes in the X-SEG co-expression network

Hub genes are excellent representatives of each co-expression module that exhibit important biological significance in system analyses. The number of edges within each node was used to calculate hubness: the more edges in a node, the higher the hubness of the gene. The edge numbers were positively associated with the absolute *k*_ME_ values. *k*_ME_, characterized as the eigengene-based connectivity for each gene, was calculated using Pearson’s correlation coefficients between the expression level and module eigengenes. The *k*_ME_ values and edge numbers were used to assign hub genes (Xie et al., 2019). The top 50 or 100 hub nodes were then identified using the maximal clique centrality (MCC) technique, which was ranked using the Cytoscape plugin cytoHubba (Chin et al., 2014). Hub genes were subjected to GO and KEGG analyses, and their homologous counterparts were identified in the *Arabidopsis* and *Populus* databases by combining these results.

### 2.8 Evaluation of BR content

From May to August 2020 (14 May, 15 May, 15 July, and 13 August), stem samples were collected from *F. mandshurica* (M8) and *F. mandshurica* × *F. sogdiana* (1601) plants 1.3 m above the ground level to measure their corresponding BL levels *in vivo*. The stem tissues were then cut into small pieces, and the phloem peel was removed before phosphate-buffered saline (PBS; pH 7.4) was added. Subsequently, the samples were immediately frozen in liquid nitrogen. BR levels were determined using a plant BR (brassinolide) ELISA Kit (Beijing Chenglin Biotechnology Company, Beijing, China), with each sample consisting of three biological replicates (n = 3).

### 2.9 Vector construction and transient expression of *FmCPD* in *F. mandshurica*

Full-length *FmCPD* cDNA was cloned and inserted into the pROKII-GFP vector under the control of the CaMV 35S promoter, which acted as an overexpression construct for *FmCPD* (OE-*FmCPD*). An empty pROKII-GFP plasmid was alternatively used as the control. The recombinant plasmid, 35S::*FmCPD*-GFP, was transformed into the *Agrobacterium tumefaciens* strain GV3101 using the freeze-thaw method. Additionally, 3-week-old *F. mandshurica* plants were transformed with *FmCPD* (OE-*CPD*) to allow the transient overexpression of FmCPD, with an empty pROKII-GFP plasmid used as a control (CON). A minimum of three seedlings from each transgenic *F. mandshurica* plant were harvested, and stem samples were immediately frozen in liquid nitrogen for quantitative real-time reverse transcription-polymerase chain reaction (qRT-PCR) analysis. Therefore, each line was analyzed using three biological replicates. Single colonies of the *A. tumefaciens* GV3101 strain, harboring 35S::*FmCPD* or empty pROKII, were cultured to an OD600 of 1; these cells were then harvested using centrifugation. The transformants were suspended in 1/2 MS medium with 3% (w/v) sucrose, 150 μM of acetosyringone, and 0.01% (w/v) Tween 20 and cultured to an OD600 of 1. Next, *F. mandshurica* seedlings were immersed in the transformation solution and incubated at 25°C for 1 h. After transformation, the *F. mandshurica* seedlings were co-cultured for 24, 36, and 48 h. Subsequently, the seedlings were washed once for 1 min in sterilized water before being planted vertically on 1/2 MS solid medium. Whole plant material was collected after growth on the plates, and RNA was extracted.

### 2.10 Quantification and validation of gene expression levels

A total of 15 DEGs were selected for qRT-PCR to validate the reliability of the prior RNA-seq analysis. First, first-strand cDNA was synthesized from 0.5 μg of the purified RNA using a Reverse Transcriptase kit (TaKaRa, Dalian, China). qRT-PCR was then performed using TransStart Top Green qPCR SuperMix (TransGen Biotech, Beijing, China), with PCR conditions according to the manufacturer’s instructions. *F. mandshurica* α-tubulin (*FmTU*) served as an internal control. The relative abundance of the transcripts was determined using the 2^−ΔΔCT^ method. To ensure accuracy, the experiment was conducted three times. All primers used in this study are listed in **Supplementary Table S8**.

### 2.11 Statistical analyses

The data were evaluated for statistical significance (p-value) using a one-way Student’s t-test, with all the tests being two-tailed. The data were normalized, and all samples were normally distributed with homogeneity of variance.

## 3 Results

### 3.1 Differences in the xylem development of *F. mandshurica* (M8) and *F. mandshurica* × *F. sogdiana* (1601)

**Figure 1:**
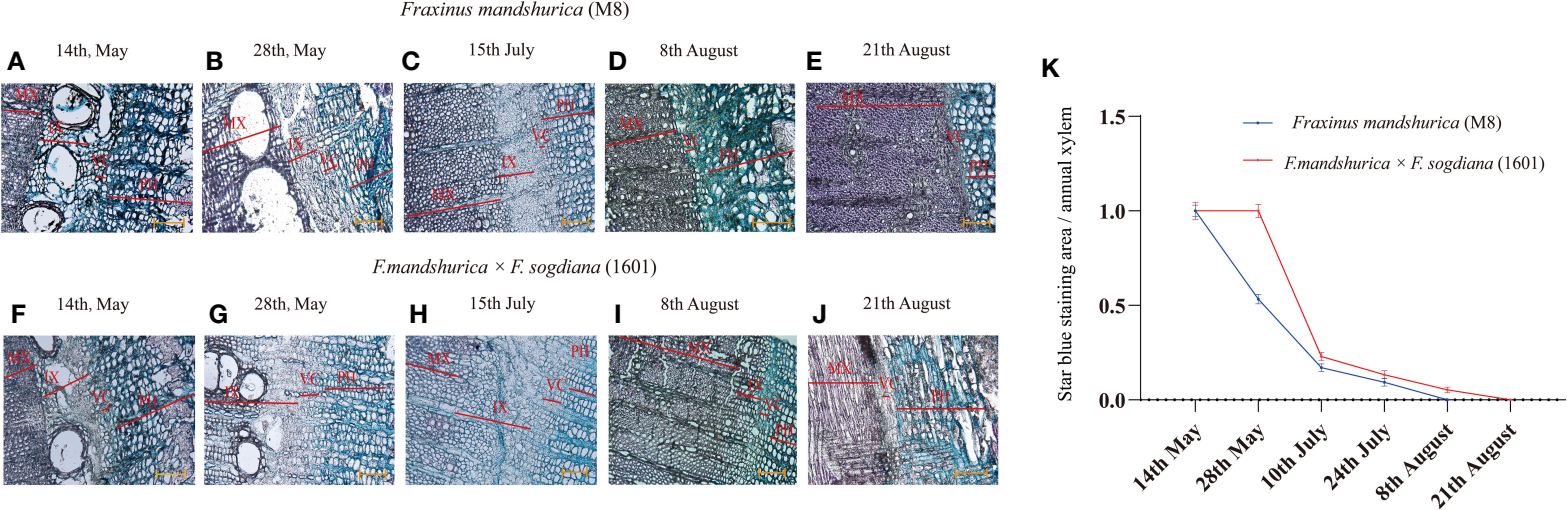
Astra/safranin staining of transverse sections of *Fraxinus mandshurica* (M8) and *F. mandshurica* × *Fraxinus sogdiana* (1601) from active to dormant stage. **(A–J)** Cytological changes in the vascular cambium, phloem, and xylem on 15 May **(A, F)**, 30 May **(B, G)**, 20 June **(C, H)**, 8 August **(D, I)**, and 21 August 2020 **(E, J)**. **(K)** Ratio of star-blue stained area to annual xylem. Ph, phloem; Vc, vascular cambium; IX, immature xylem; MX, mature xylem. Values are means ± SD (n = 5). Scale bars: **(A–J)** = 100 μm.

**Figure 2:**
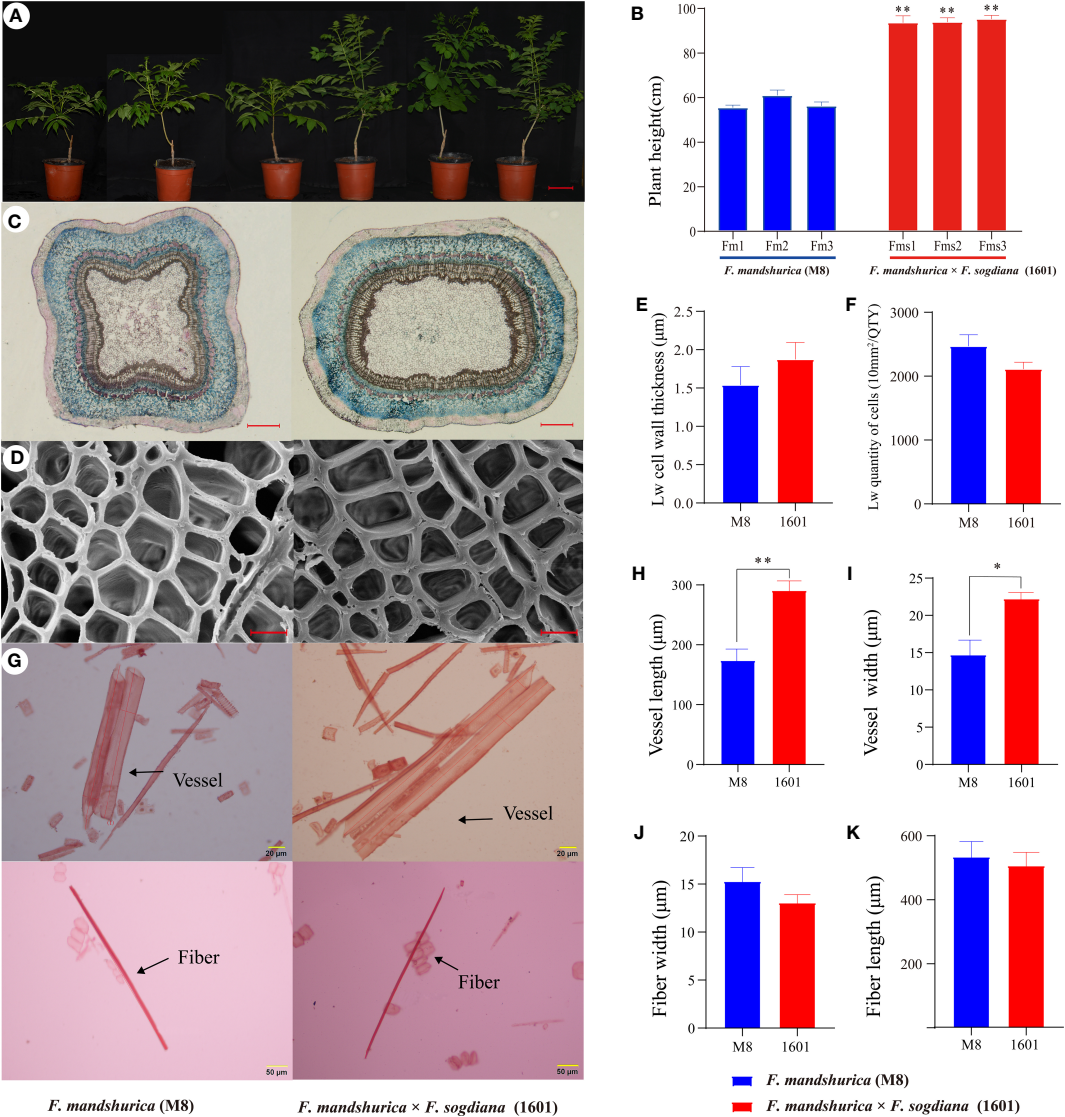
Properties of wood in *Fraxinus mandshurica* (M8) and *F mandshurica* × *Fraxinus sogdiana* (1601) during latewood development stage. **(A)** Plant morphological phenotypes of 1-year-old M8 (left) and 1601 (right). **(B)** Measurement of plant height. **(C)** Wood section of xylem morphological in M8 (left) and 1601 (right). **(D)** Scanning electron microscopy (SEM) analysis of xylem in the later wood sections of M8 (left) and 1601 (right). **(E)** Cell wall thickness and **(F)** quantity of cells in the unit area (10 mm^2^/QTY) in later wood. **(G)** Fibers and vessels from M8 and 1601. **(H–K)** Length and width of vessels and fiber cell respectively; the values were means ± SE of 400 independent fibers/vessels from M8 and 1601. **(C–K)** The experimental materials were taken from the xylem of 3-year-old M8 (left) and 1601 (right). Significance was determined by Student’s t-test (*p < 0.05 and **p < 0.01). Scale bars: **(A)** = 10 cm; **(C)** = 1 mm; **(D)** = 10 μm; **(G)** = 20/50 μm.

**Figure 3:**
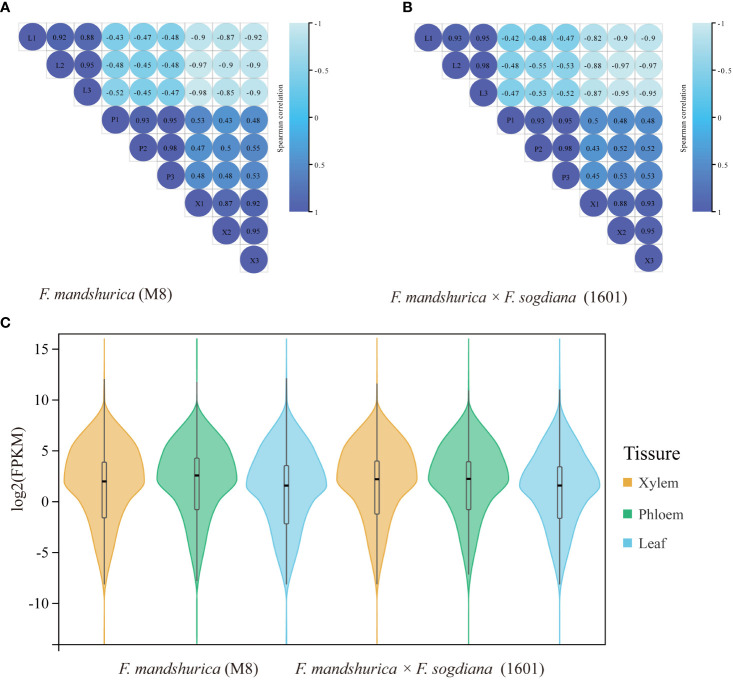
Correlation analysis and the gene expression distributions of different tissues (X, xylem; CP, phloem; L, leaf) in the two tree groups. **(A, B)** Correlations among different tissues and replicates of *Fraxinus mandshurica* (M8) and *F mandshurica × Fraxinus sogdiana* (1601). **(C)** Gene expression distribution of different tissues in two tree groups. Violin plots appear in the center. The expression levels of the genes were standardized by the log2 (FPKM) algorithm. FPKM, fragments per kilobase million.

**Figure 4:**
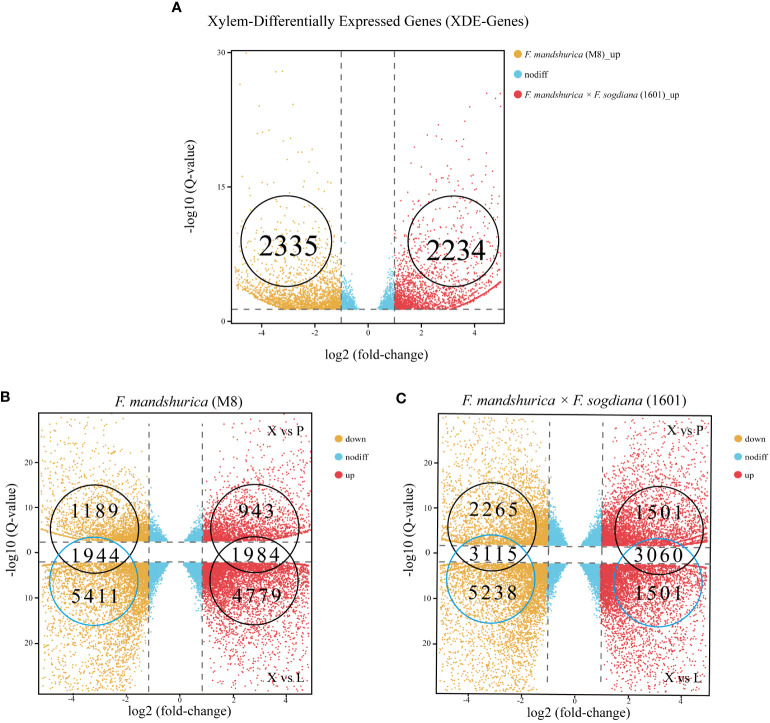
Distributing up and downregulated DEGs in *Fraxinus mandshurica* (M8) and *F mandshurica × Fraxinus sogdiana* (1601). **(A)** Upregulated and downregulated X-DEGs between M8 and 1601. **(B)** Upregulated and downregulated X-SEGs in M8. **(C)** Upregulated and downregulated X-SEGs in 1601. In panels **(B, C)**, X vs. P represents the differential expression analysis between the xylem and phloem; likewise, X vs. L represents the differential expressed analysis between the xylem and leaf. The vertical dotted lines correspond to the log2 (fold change) = ± 1, while the horizontal dotted lines are positioned at log10 (q-value) = ± 1.2 (q-value = 0.05). The genes at the intersection of two groups are the upregulated and downregulated genes in xylem tissue. DEGs, differentially expressed genes; X-DEGs, xylem differentially expressed genes; X-SEGs, xylem specifically expressed genes.

**Figure 5:**
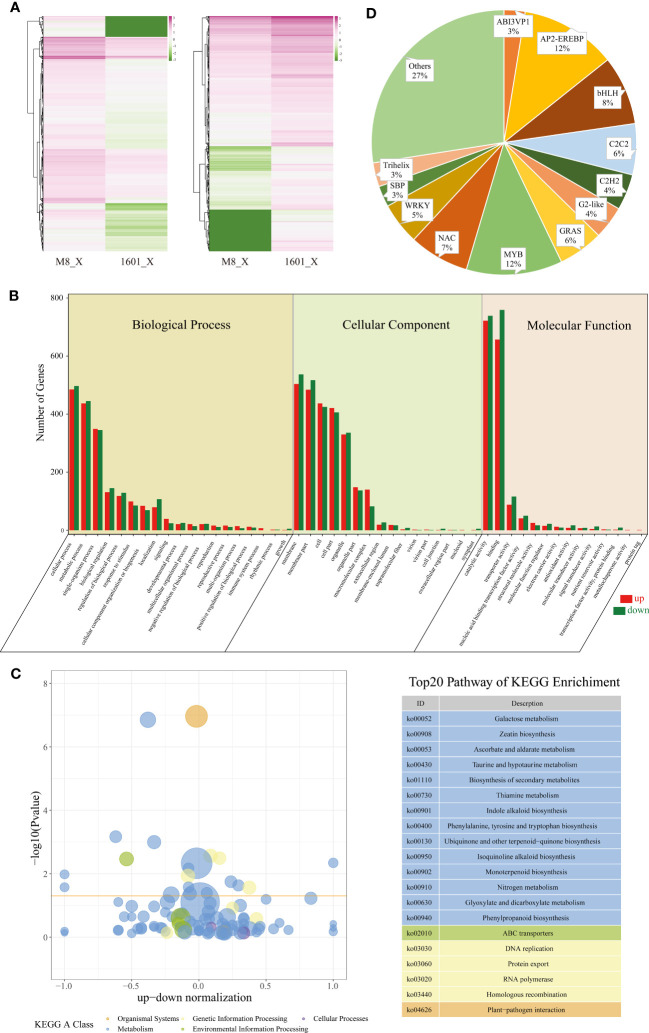
X-DEGs between *Fraxinus mandshurica* (M8) and *F mandshurica × Fraxinus sogdiana* (1601). **(A)** Heatmap of up- and downregulated X-DEGs in M8 and 1601. Upregulated in M8 (left) and upregulated in 1601 (right). **(B)** Gene Ontology (GO) classification of X-DEGS; GO was summarized into three main categories: biological process (left panel), cellular component (middle panel), and molecular function (right panel). The number percentage (Y-axis) of genes and GO categories (X-axis) are also shown. **(C)** KEGG analysis resulted according to the Rich factor, with the top 20 pathways chosen for visualization purposes. **(D)** Distributions of transcription factors sorted into different families. X-DEGs, xylem differentially expressed genes; KEGG, Kyoto Encyclopedia of Genes and Genomes.

**Figure 6:**
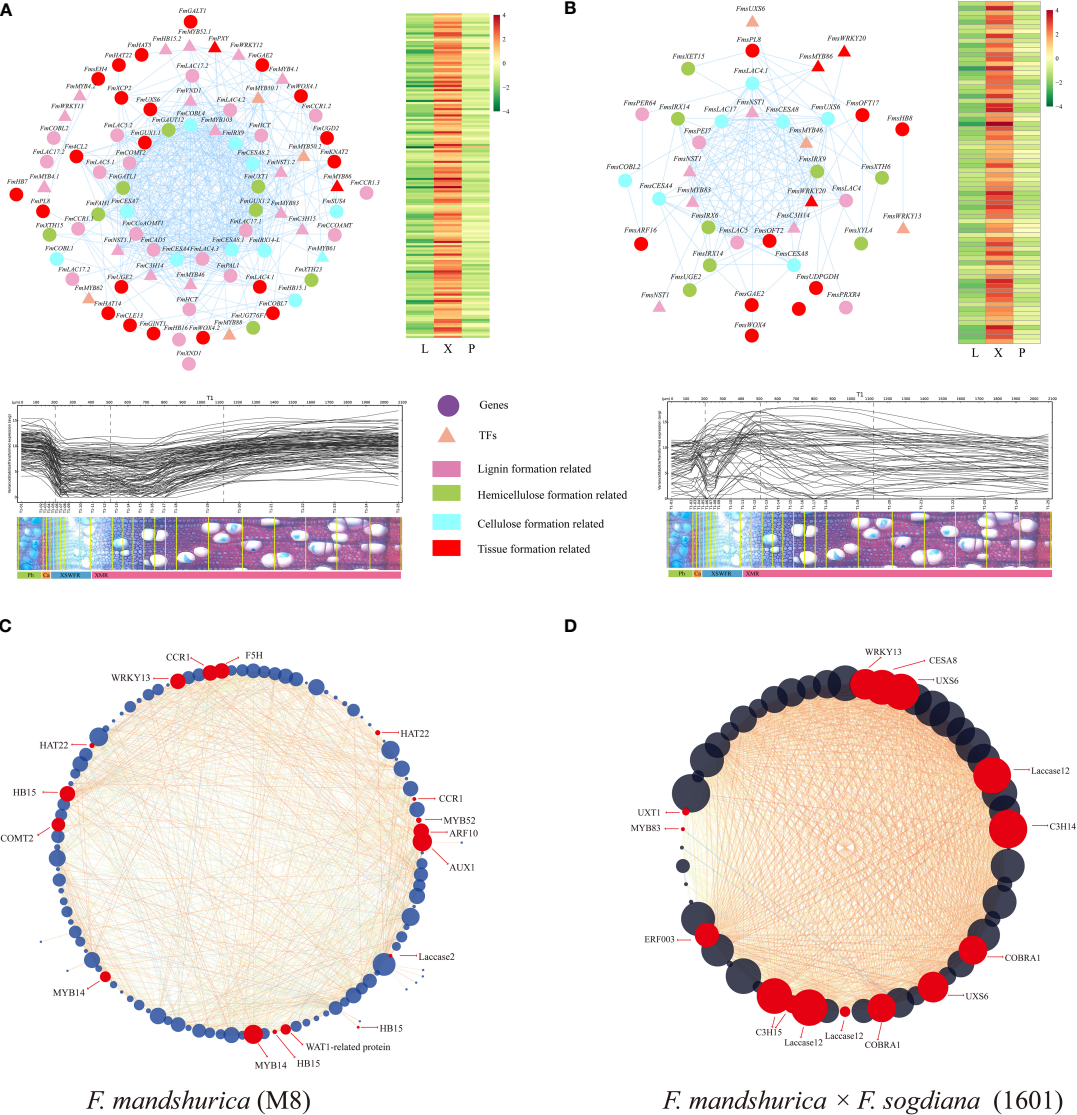
Co-expression network of wood formation-related genes in the two tree groups. **(A, B)** Network for wood formation-related genes in *Fraxinus mandshurica* (M8) and *F mandshurica* × *Fraxinus sogdiana* (1601), respectively. The heatmaps (right) and gene expression abundance curves (below) depict the corresponding genes in each co-expression network. The scales above the expression curves indicate the slice position; the scales under the expression curves indicate the number of slices. Gene expression abundance curves were derived and drawn using an online tool (http://aspwood.popgenie.org). **(C, D)** Co-expression network of xylem tissue-specific hub genes in two tree groups; red dots represent genes involved in xylem formation. The co-expression network map was drawn by Cytoscape (version 3.9.0), maximal clique centrality (MCC) program was selected, and the genes with *k*_ME_ values in the top 100 **(A, B)** and 50 **(C, D)** were selected for visual analysis, respectively.

**Figure 7:**
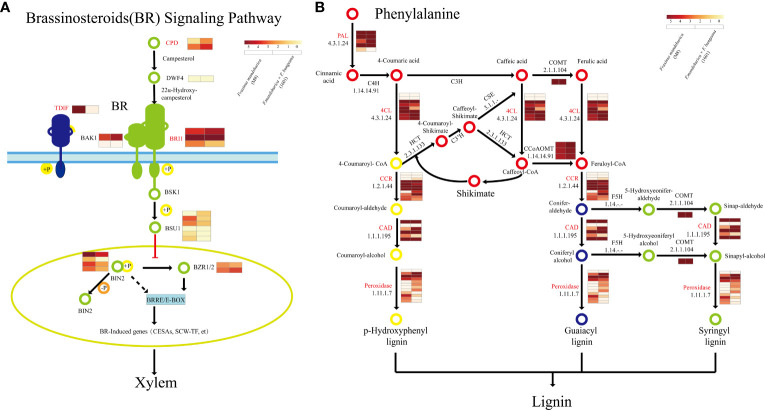
Wood formation-related pathways of *Fraxinus mandshurica* (M8) and *F mandshurica* × *Fraxinus sogdiana* (1601). **(A)** Brassinosteroid synthesis and signaling pathway. **(B)** Lignin synthesis pathway. Genes in red are both X-DEGs and X-SEGs in M8 and 1601. X-DEGs, xylem differentially expressed genes; X-SEGs, xylem specifically expressed genes.

**Figure 8:**
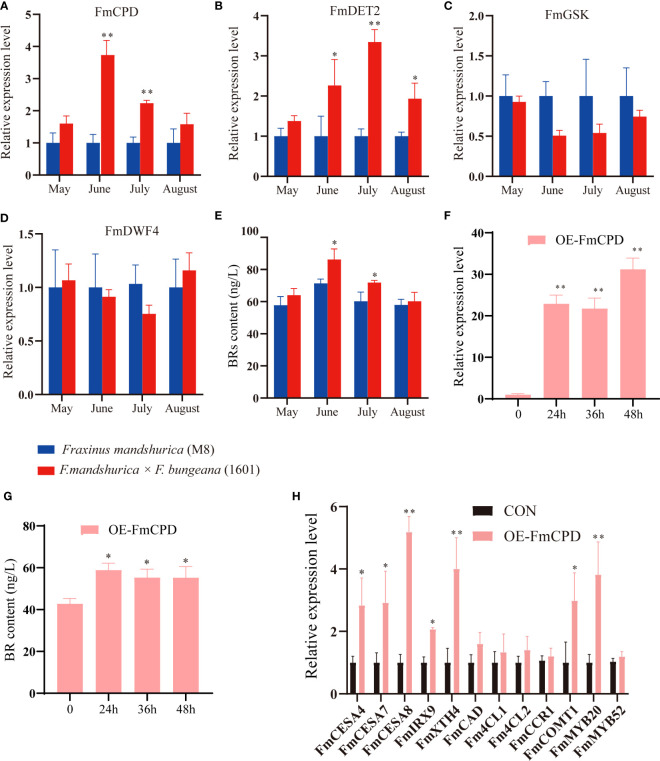
Brassinosteroids (BRs) affected the xylem development of *Fraxinus mandshurica*. **(A–D)** The relative expression levels of four genes involved in BR synthesis and signaling pathway from May to August between the xylem of *F. mandshurica* (M8) and *F. mandshurica × Fraxinus sogdiana* (1601). **(E)** The determination of BR content from May to August between the xylem of M8 and 1601. **(F)** FmCPD transcript levels in *F. mandshurica* with transient overexpression *FmCPD*. the expression of *FmCPD* was determined by qRT-PCR. CON (0 h), plants transformed with empty 35S::pROKII-GFP; OE, plants transformed with pROKII-*FmCPD*-GFP for overexpression of *FmCPD*. **(G)** The determination of BR content between OE-CPD and CON (0 h). **(H)** Expression analysis of genes and TFs involving the lignin, cellulose, and hemicellulose presents synthesis. Values in the figure represent the means ± SE of three replicates. Significance was determined by Student’s t-test (*p < 0.05 and **p < 0.01). TFs, transcription factors.

**Figure 9:**
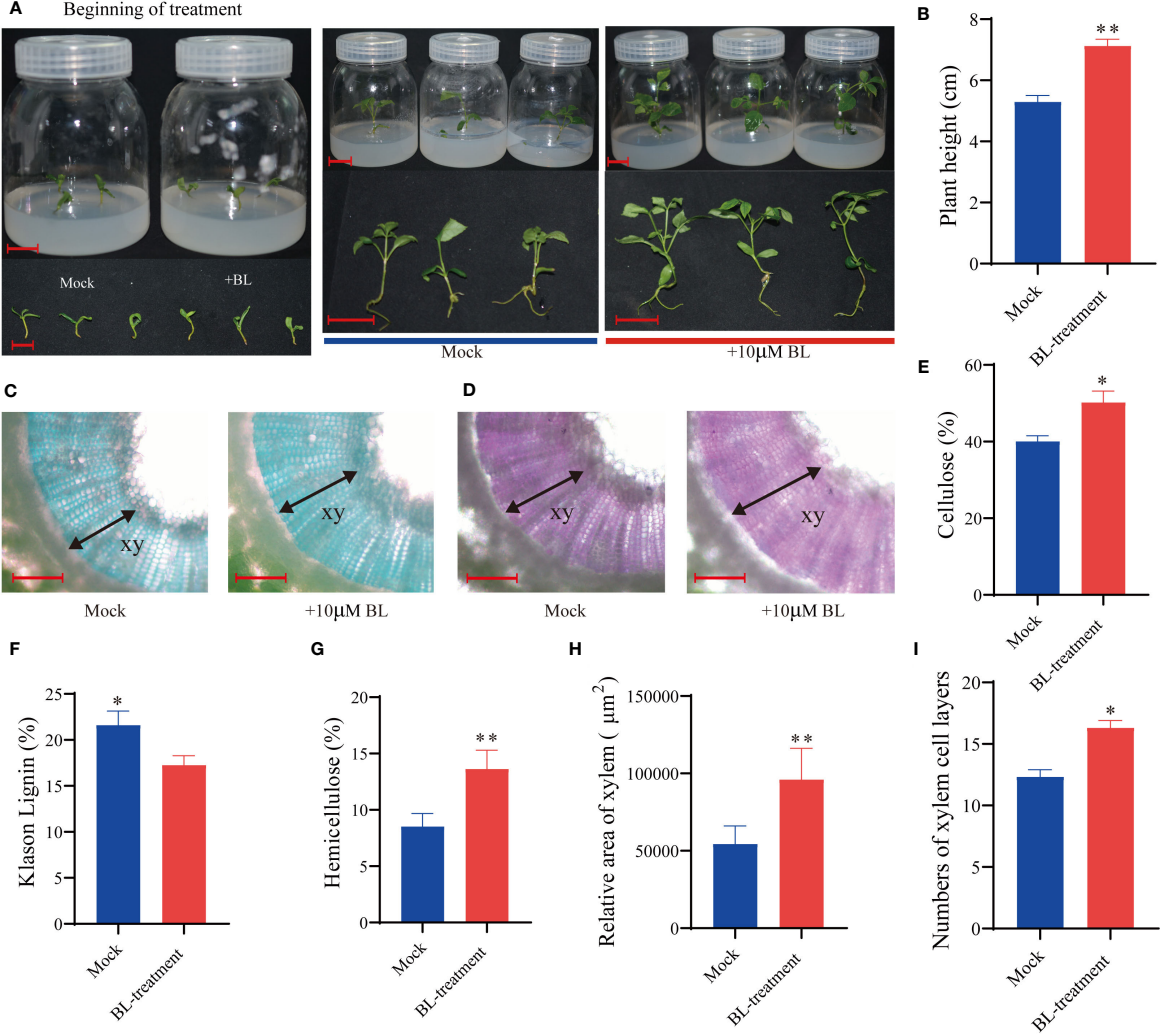
Exogenous application of BL induces stem growth and influences xylem formation in Fraxinus mandshurica. **(A, B)** Growth promotion of BR-treated (10 mM of BL) stems with the effects observed after 3 weeks. **(C, D)** Fast green/safranin-stained hand cross-sectional views of a stem after exogenous application of BL. Measurement of cellulose content **(E)**, lignin content **(F)**, and hemicellulose content determination **(G)**. The number of cell layers and the measurement of the relative area of xylem between BL-treated and mock plants **(H, I)**. G and H were investigated using cross-sectional views. A total of 20 spots of 80 mm2 were selected on a picture from three to five sections for each application. Significance levels are indicated by one asterisk (*p < 0.05 and **p < 0.01). Scale bars: **(A)** = 1.50 cm; **(B)** = 200 mm; **(C, D)** =200μm.

The xylem developmental patterns of *F. mandshurica* (M8) and *F. mandshurica* × *F. sogdiana* (1601) were investigated over a period of 4 months. Transverse section analysis revealed that the cambial zone formed a narrow band with thin primary cell walls (**Figures 1A–J**). On 14 May, both M8 and 1601 variants exhibited an onset of xylem development, characterized by a loose internal structure and increased vessel formation; this indicated that both variants were in the early stages of wood formation, which is typical of the active growth stage of ring-porous wood trees (**Figures 1A, F**). From 28 May (**Figures 1B, G**) to 15 July (**Figures 1C, H**), unlike the mature xylem that was stained red by safranin, the number of cambium and secondary xylem cells increased, as measured by astra blue in M8 and 1601; this indicated that these plants were in the active stage of growth. By the third time point, the cambial zone had receded (**Figures 1G, H**), and the newly formed secondary xylem (forming latewood) had begun to thicken in both M8 and 1601 variants, indicating that both tree groups had reached the latewood development stage. Nonetheless, the incomplete lignification region of M8 was smaller than that of 1601, indicating that M8 had a higher lignification rate than 1601. Finally, from 8 to 21 August (**Figures 1D, E, I, J**), the cambial cells shrank to form a thin band, and the immature secondary xylem gradually disappeared, thereby forming wood observed in the late growing season. During this period, the xylem of 1601 retained a blue incomplete lignification region, whereas the xylem of M8 had been completely lignified, and wood development had ended. This demonstrated that the radial development cycle of the 1601 xylem was 2 weeks longer than that of M8 (**Figure 1K**).

### 3.2 Properties of wood in *F. mandshurica* (M8) and *F. mandshurica* × *F. sogdiana* (1601)

Throughout this study, we examined the differences in the radial xylem development of *F. mandshurica* (M8) and *F. mandshurica* × *F. sogdiana* (1601); overall, *F. mandshurica* × *F. sogdiana* (1601) (93.8 cm) exhibited a 47% increase in growth height compared to M8 (57.1 cm) (**Figures 2A, B**), indicating significant variations in growth patterns. During the latewood developmental stage, cross-sectional shape analysis using histochemical staining revealed substantial differences in the xylem morphology of each group, with M8 exhibiting a square-shaped cross-section, whereas 1601 possessed an oval-shaped cross-section (**Figure 2C**). Scanning electron microscopy (SEM) analysis demonstrated that the number of xylem cells was similar in M8 and 1601, but 1601 cells possessed thicker vessel cell walls than M8 cells. Further, tissue segregation analysis revealed that 1601 exhibited a 67% increase in vessel width and a 47% increase in vessel length when compared to M8; however, no significant differences in fiber width and length were observed (**Figures 2G–K**). Chemical analysis of the SCW components indicated that variant 1601 contained higher levels of cellulose (increased by 14%) and lower levels of Klason lignin (decreased by 11%) than M8 (**Table 1**). These findings demonstrate that M8 and 1601 exhibited significant differences in cellular structure and wood chemical composition during the latewood development stage.

**Table 1:** Cell wall composition analysis of the stems in *Fraxinus mandshurica* (M8) and *F. mandshurica × Fraxinus sogdiana* (1601).

Cell wall component	*F. mandshurica* (M8)			*F. mandshurica × F. sogdiana* (1601)		
Sample	L1	L5	L13	L9	L11	L15
Lignin (mg/100 mg, %)						
Acid-insoluble	25.58 ± 0.52	24.75 ± 0.58	25.12 ± 0.37	21.60 ± 0.49	22.27 ± 0.52	22.03 ± 0.58*
Acid-soluble	1.39 ± 0.23	2.01 ± 0.25	1.92 ± 0.13	2.15 ± 0.15	1.87 ± 0.19	1.61 ± 0.25
Total	26.97 ± 0.75*	26.76 ± 0.83*	27.04 ± 0.50*	23.85 ± 0.64	24.2 ± 0.71	22.64 ± 0.83
Cellulose (mg/100 mg, %)						
	42.41 ± 0.12	41.09 ± 0.35	41.70 ± 0.31	48.21 ± 0.25*	46.93 ± 0.35*	52.21 ± 0.45*
Hemicellulose (mg/100 mg, %)						
	12.04 ± 0.31	11.90 ± 0.55	12.88 ± 0.31	10.44 ± 0.37	11.24 ± 0.25	11.52 ± 0.55

Plant materials were prepared from the stems of 3-year-old plants and determined by Klason methods. Error bars represent ± SD from three biological repeats. Asterisks indicate significant differences between F. mandshurica (M8) in comparison to F. mandshurica × F. sogdiana (1601) Significance was determined by Student’s t-test (*p < 0.05). The unit is mg per 100 mg.

### 3.3 RNA-seq global analysis of *F. mandshurica* (M8) and *F. mandshurica* × *F. sogdiana* (1601)

RNA-seq was performed on the xylem, phloem, and leaf tissues of *F. mandshurica* (M8) and *F. mandshurica* × *F. sogdiana* (1601). Before formal data analysis, the gene expression levels and correlation (using Pearson’s r) among the 18 samples (different replicates and tissue types) were analyzed. The tests confirmed a strong correlation between the three replicates of the same tissue in each species (r values >0.8; **Figure 3A**), indicating that their corresponding transcriptome data were suitable for further analysis. Overall gene expression levels were similar between M8 and 1601, indicated by the violin plot of their corresponding FPKM values (**Figures 3B, C**).

### 3.4 Identification of X-DEGs and X-SEGs

In total, 57.96 Gb and 57.85 Gb of clean reads were produced from *F. mandshurica* (M8) and *F. mandshurica* × *F. sogdiana* (1601), respectively. Over 91% of the reads were obtained from the Q30 level. In M8, we obtained 42.97 million, 43.04 million, and 42.77 million clean reads in the xylem, phloem, and leaf tissues, respectively; further, 42.69 million, 42.96 million, and 42.92 million clean reads were obtained from the xylem, phloem, and leaves of 1601, respectively. Reads from M8 and 1601 were all mapped to a reference genome (http://www.ashgenome.org/). The mapping rates for the xylem, phloem, and leaf tissues were 76.29%, 80.71%, and 77.93%, respectively, for M8 and 77.14%, 79.08%, and 76.40%, respectively, for 1601 (**Supplementary Table S1**). Finally, we obtained 44,561 genes in the xylem, 47,465 in the phloem, and 48,091 in the leaves of M8, alongside 34,629 in the xylem, 22,986 in the phloem, and 23,233 in the leaves of 1601 (**Supplementary Table S1**).

A total of 4,589 DEGs were observed between the M8 and 1601 xylem samples (p-value <0.05 and log2 ratio ≥1, ratio = 1601X/M8X); 2,234 of these X-DEGs were upregulated, while the other 2,355 X-DEGs were downregulated in 1601 (**Figure 4A**; **Supplementary Table S2A**). To understand the difference in RNA expression between the three different tissues of M8 and 1601, an X-SEG analysis was also conducted. For M8, 6,763 upregulated X-SEGs and 7,355 downregulated X-SEGs were observed in the X vs. L group; 2,927 upregulated X-SEGs and 3,133 downregulated X-SEGs were observed in the X vs. CP group (**Figure 4B**; **Supplementary Tables S3A, B**). For 1601, the following were obtained: 6,775 upregulated X-SEGs and 8,353 downregulated X-SEGs in the X vs. L group and 4,561 upregulated X-SEGs and 5,380 downregulated X-SEGs in the X vs. CP group (**Figure 4C**; **Supplementary Tables S4C, D**). Finally, by considering the overlapping intersection of X vs. L and X vs. CP, the following were obtained: 3,928 (1,984 upregulated and 1,944 downregulated) X-SEGs in M8 (**Figure 4B**) and 6,175 (3,060 upregulated and 3,115 downregulated) X-SEGs in 1601 (**Figure 4C**).

### 3.5 Analysis and functional classification of X-DEGs

Next, we analyzed gene expression levels and classified the X-DEGs identified among the six samples. Overall, 4,589 genes were differentially expressed in the xylem of *F. mandshurica* (M8) (2,355 X-DEGs upregulated) and *F. mandshurica* × *F. sogdiana* (1601) (2,234 X-DEGs upregulated), as depicted in a heatmap (**Figure 5A**).

To determine the relationship between X-DEGs and wood traits in M8 and 1601, we performed a GO enrichment analysis of 46 functional groups belonging to the BP, CC, and MF categories. The top three GO terms were “retrotransposon nucleocapsid”, “nuclear part”, and “Smc5–Smc6 complex” for the CC category; “DNA integration”, “DNA metabolic process”, and “nucleic acid metabolic process” for the BP category; and “RNA-directed DNA polymerase activity”, “ADP binding”, and “DNA polymerase activity” for the MF category (**Figure 5B**; **Supplementary Table S2B**). We also conducted a KEGG pathway enrichment analysis of the M8 and 1601 X-DEGs, which elucidated 133 corresponding pathways; 20 of these pathways were determined to be significantly enriched in the xylem (**Figure 5C**; **Supplementary Table S2C**) (p < 0.05).

Based on wood composition changes observed between M8 and 1601, we identified 83 X-DEGs (33 upregulated and 50 downregulated) involved in lignin cellulose biosynthesis. Among the X-DEGs, 63 were involved in phenylpropanoid biosynthesis; this included phenylalanine ammonia-lyase (*PAL*), cinnamyl alcohol dehydrogenase (*CAD*), peroxidase, 4-coumarate coenzyme A ligase (*4CL*), cinnamoyl-CoA reductase (*CCR*), coniferyl-alcohol glucosyltransferase (*UGT72E*), and beta-glucosidase (**Supplementary Table S2E**). Additionally, 19 DEGs were associated with cellulose synthase. The expression levels of two genes encoding cellulose synthase-like (*CSL*) D3 and E1 changed significantly in 1601; alternatively, three genes encoding cellulose synthases, *CESA2*, *CESA3*, and cellulose synthase interactive 3 (*CSI3*), were upregulated in M8 (**Supplementary Table S2E**). Of the 83 X-DEGs, a total of 10 DEGs related to hemicellulose synthesis, primarily xyloglucan endotransglycosylase/hydrolase (*XET*), were significantly upregulated in 1601 (**Supplementary Table S2E**).

We observed differential expression between the xylem of M8 and 1601 with regard to a series of X-DEGs involved in plant hormone biosynthesis and signal transduction (**Supplementary Table S2F**). Among these genes, 38 were involved in the auxin signaling pathway, with the expression levels of 23 genes being significantly higher in M8 than in 1601. Interestingly, two indole-3-acetic acid family transcription factors (TFs) were upregulated in 1601. Moreover, 24 X-DEGs were involved in the ethylene pathway, of which 10 were upregulated in M8, and 14 were downregulated in 1601. We also identified 27 DEGs involved in the biosynthesis and signaling pathway of BRs. Two genes encoding brassinosteroid insensitive 1 (*BRI1*; F000324690 and F000009900) and two genes encoding constitutive photomorphogenic dwarf (*CPD*; F000362610 and F000110180) were highly expressed in the 1601 variant. Alternatively, *DWF4* (F000297730), *TINY* (F000123110), *CLAVATA3/ESR* (*CLE*)-related protein *TDIF* (F000010950), and shaggy-related protein kinase (*GSK*, F000131280) were highly expressed in the M8 xylem. In addition, we identified 53 DEGs involved in starch and sucrose metabolism (**Supplementary Table S2G**).

TFs are crucial regulatory genes involved in plant growth and development. We identified 277 distinctly expressed TFs that were differentially expressed between the two tree variants (q ≤ 0.05, log2 fold change >1 or <−1) (**Supplementary Table S2D**). These genes include TFs from various families, including MYB (32), AP2/ERF (32), bHLH (23), NAC (20), C2C2 (17), GRAS (15), WRKY (14), C2H2 (12), G2-like (11), trihelix (8), ABI3VP1 (7), SBP (7) LOB (6), MADS (5), ARF (4), TCP (4), and bZIP (3). Notably, two AP2/ERF genes (*ERF5*, F000249810; *ERF104*, F000244550) were highly expressed in 1601, and two MYB genes (*MYB8*, F000339870; *MYB52*, F000365430) and one NAC gene (*NAC104*, F000154890) were highly expressed in the M8 xylem (**Figure 5D**; **Supplementary Table S2D**).

### 3.6 Functional classification and WGCNA of X-SEGs

To assess and compare the functional differences of X-SEGs, we compared GO and KEGG analyses of *F. mandshurica* (M8) and *F. mandshurica* × *F. sogdiana* (1601) (**Supplementary Figure 2**; **Supplementary Table S4**). We ordered the corresponding results by p-value for KEGG analysis and selected the top 20 pathways to visualize their representative pathways. The results revealed different gene functions in the metabolic processes of M8 and 1601. Using WGCNA, we determined that the 3,928 M8 X-SEGs could be merged into four dynamic modules: blue, brown, turquoise, and yellow (**Supplementary Figure 3A**). Alternatively, 6,175 of 1601 X-SEGs were merged into six modules: black, brown, blue, green, turquoise, and yellow (**Supplementary Figure 3B**). We then used GO analysis to further describe the functions of the genes within each module (**Supplementary Figure 3**; **Supplementary Table S6**).

After conducting WGCNA for each selected module of each tree variant, we visualized the gene co-expression network by selecting the top 1,000 gene pairs ordered by the edge weight coefficient. We then performed a follow-up GO analysis for each module to describe the function of the genes within each module. Only the top 10 GO numbers in BP have been illustrated graphically. For M8, GO analysis indicated that more wood formation-related genes were clustered within the blue module. Among the top 10 GO terms, GO:0009808, GO:0046274, and GO:0046271 were associated with lignin biosynthesis. Alternatively, GO:0071554, GO:0042546, GO:0009698, and GO:0046271 were associated with cell wall organization and phenylpropanoid metabolic processes. Further, GO:0071365 and GO:0032870 were associated with cellular responses to hormonal stimuli (**Supplementary Figure 4**; **Supplementary Table S6A-H**). For 1601, the top 10 GO terms, including cell wall biogenesis (GO:0042546) and lignin catabolic processes (GO:0046274), were significantly enriched in the black module, which is also a wood formation-related module (**Supplementary Figure 5**; **Supplementary Table S6I-T**). By combining the GO and KEGG results, we identified that the most critical modules involved in lignin synthesis and SCW formation in M8 and 1601 were turquoise and black, respectively.

Next, we conducted a comparative analysis between *F. mandshurica* wood formation genes and homologous genes in *Arabidopsis* and *Populus* spp.; this revealed distinct patterns in phenylpropanoid metabolism, BR synthesis, and signaling pathways (**Supplementary Table S5**). Specifically, we established that phenylpropanoid metabolism, BR synthesis, and signaling pathway patterns differed in *F. mandshurica* (**Supplementary Table S5**). Next, we employed the MCC algorithm in CytoHubble (Cytoscape version 3.9.0) to screen for hub genes with high *k*_ME_ values; we then visualized the top 100 gene nodes for the most critical modules in M8 and 1601. We verified the co-expression relationships of all key module genes forming the network using the AspWood web resource (http://aspwood.popgenie.org) (**Supplementary Table S7**). Notably, the genes within the co-expression networks exhibited actual co-expression relationships across xylem tissue (≥300 μm) for both groups (**Figures 6A, B**), indicating the robustness and effectiveness of our co-expression networks for establishing an understanding of these mechanisms.

Furthermore, we observed notable similarities in the expression patterns of *F. mandshurica* (M8) and *F. mandshurica* × *F. sogdiana* (1601), as evidenced by the presence of laccase family-related genes and genes encoding *WRKY13* (F000342230 and F000268170) within the core control network. However, clustered hub genes were significantly different. In *F. mandshurica* (M8), we selected 24 co-expressed genes involved in xylem and secondary wall formation for further visual analysis (**Figure 6C**), including CAN (*HB15*; F000255150, F000341940, and F000043600) from the HD zip III family. *MYB52* (F000197340) and walls are thin 1 (*WAT1*; F000217510) were determined to be closely associated with SCW synthesis. Moreover, functional genes that are involved in lignin synthesis via the phenylpropanoid pathway were also identified, including *F5H* (F000213810), *CCR1* (F000387280), and *COMT2* (F000085420) (**Figure 6C**; **Supplementary Table S7A**). In *F. mandshurica* × *F. sogdiana* (1601), the top hub genes involved in lignin cellulose synthesis and cell wall deposition included *C3H14* (F000343280), *C3H15* (F000168920), *MYB83* (F000027510), *COBRA1* (F000353330 and F000300000), and *CESA8* (F000276400) (**Figure 6D**; **Supplementary Table S7B**). Furthermore, the co-expression networks of both tree groups identified all the genes involved in xylem development within the key modules, most of which were involved in the synthesis of lignin, cellulose, and hemicellulose. Interestingly, there were group-specific genes in each of the two tree groups; for example, *MYB103* (F000281250) was specifically expressed in the xylem of 1601, and *XCP2* (Xylem Cysteine Protease 2, F000244210) was specifically expressed in the xylem of M8 (**Supplementary Table S7**). Our WGCNA results highlight the marked differences in the top-level control of xylem development in M8 and 1601 trees and the potential impact of tree-specific genes in latewood development stages. Additionally, we established that the qRT-PCR results for 15 genes related to lignin synthesis were consistent with those obtained in the RNA-seq dataset (**Supplementary Figure 1**).

### 3.7 Combined analysis of X-DEGs and X-SEGs to analyze wood formation-associated pathways

Here, we focused on three key pathways involved in wood formation: phenylpropanoid biosynthesis, cellulose biosynthesis, and BR synthesis and signaling. We initially associated each pathway with corresponding X-DEGs and X-SEGs based on the KEGG results and noted that most of these genes were activated during the same step processes in both tree groups. Specifically, in the BR synthesis and signaling pathways (**Figure 7A**), five genes were highly expressed in the xylem of all three groups, including *BRI1*, *SPL16*, brassinosteroid insensitive 1-EMS-suppressor (*BES1*), *BES1/BZR1* homologs *4* (*BEH4*), and *BRI1* kinase inhibitor 1 (*BKI1*) (**Supplementary Table S5**). Notably, in 1601, *FmCPD* (F000362610), which is most similar to the *Arabidopsis AtCPD* gene, *BRI1*, and *DEETIOLATED2 (DET2)* were upregulated, whereas *DWF4* and GSK3 protein kinases were upregulated in M8 (**Supplementary Table S5**).

Although both tree groups harbored homologous genes involved in the phenylpropanoid biosynthesis pathway, their expression patterns were not identical. In the phenylpropanoid biosynthesis pathway (**Figure 7B**), *PAL1* (F000026370) was upregulated 4.85-fold in the M8 xylem, and four genes encoding phenylalanine ammonia lyase (*PAL*) were specifically expressed in M8. Conversely, only one PAL gene was specifically expressed in the xylem of variant 1601. In addition, five genes encoding 4CL were specifically expressed in M8, of which *4CL2*(F000105140) was upregulated 2.37-fold. Further, two genes encoding *CAD*, including *CAD1* (F000265860), were specifically expressed and upregulated 4.85-fold in the xylem of 1601. The number of xylem-specific genes encoding *CCoAOMT1* and *CCR* was determined to be 3 and 6 in M8, respectively, with a similar number in 1601, one of which was *CCR2* (F000220480) (**Supplementary Tables S2**, **S5A, B**).

### 3.8 BRs promote wood development in *F. mandshurica*

BRs play a vital role in wood development. Therefore, we compared the expression of BR synthases and signal conduction genes in the xylem tissues of *F. mandshurica* (M8) and *F. mandshurica* × *F. sogdiana* (1601) using qRT-PCR (**Figures 8A–D**). Our findings indicated that from May to August, the expression levels of two BR synthesis genes, *FmCPD* and *FmDET2*, were upregulated in 1601; however, no significant changes were observed in *FmDWF4* expression. Additionally, BR expression suppressed signal conduction genes, with GSK3 being downregulated in 1601 (**Figure 8C**). These results indicate higher levels of BRs than those in M8. To validate our hypothesis, we examined the xylem BR content of M8 and 1601 from May to August. The results showed that the BR content in the 1601 xylem increased significantly (**Figure 8E**), whereas it remained low at approximately 20% in the M8 xylem during June and July. Ultimately, this higher BR activity in 1601 may explain the differences in wood traits between these two tree variants.

To determine whether transient overexpression of the *FmCPD* gene in *F. mandshurica* was successful, we evaluated *FmCPD* expression in CON and OE plants using qRT-PCR. The highest expression level in the OE-*FmCPD* plants was 31.22 times higher than that in the CON plants at 48 h (**Figure 8F**). These results demonstrate that we successfully overexpressed *FmCPD* in transgenic plants. Therefore, 48 h was selected as the transfer time for the subsequent experiments. Notably, the BR content was measured during the transient overexpression of *FmCPD*; these results indicated that the BR content reached a maximum within 24 h of *FmCPD* overexpression (**Figure 8G**). We further examined the expression of SCW biosynthesis-related genes in the OE-*FmCPD* transgenic lines (**Figure 8H**). As expected, three cellulose biosynthetic genes (*FmCESA4*, *FmCESA7*, and *FmCESA8*) and two xylan biosynthetic genes (*FmIRX9* and *FmXTH4*) were significantly upregulated in transgenic plants, while five lignin synthesis-related genes (*FmCAD*, *Fm4CL1*, *Fm4CL2*, *FmCCR1*, and *FmCOMT1*) were slightly upregulated. The expression levels of SCW-associated TFs were also determined. Specifically, in the OE-*FmCPD* transgenic lines, *FmMYB20* was determined to be significantly upregulated, and *FmMYB52* was significantly downregulated.

Alternatively, exogenously applied BL may also significantly affect growth and SCW biosynthesis in the xylem of *F. mandshurica*. After 3 weeks of BL application, *F. mandshurica* seedling growth significantly accelerated (**Figures 9A, B**), cellulose and hemicellulose content increased, and xylem lignin content decreased (**Figures 9E–G**). Moreover, the fast green/safranin dye staining results of the hand-cut section indicated that the development of the secondary xylem in *F. mandshurica* seedlings was promoted by BL treatment (**Figures 9C, D**); further, the xylem area expanded (**Figure 9H**), and the number of xylem cell layers (16 layers) increased in the BL-treated plants (**Figure 9I**) compared to the mock control plants, with only 12 xylem cell layers being observed in the mock-treated plants (**Figure 9I**).

## 4 Discussion

The primary goal of this study was to investigate differences in wood properties and the potential regulators of these differences in *F. mandshurica* (female parent, M8) and the interspecific hybrid *F. mandshurica* × *F. sogdiana* (1601). Through interspecific hybridization between female *F. mandshurica* (M8) and male *F. sogdiana* (2-1), we obtained a novel hybrid clone ‘1601’ (*F. mandshurica* × *F. sogdiana*). The paternal species (2-1) used in this study has not yet been successfully introduced into northern China (Heilongjiang Province); therefore, it was not considered in this study. By tracking the developmental process and wood properties of the hybrid clone in comparison to the maternal M8 variant, we observed a 2-week extension in the growth cycle, alongside an increased vessel length and width in 1601; further, the growth rate of 1-year-old 1601 clone was found to be particularly accelerated. In 1601, the cellulose content of the wood was also significantly greater than that in M8, while the lignin content was significantly lower (**Figures 2A–K**). When considering the ecological and economic value of *F. mandshurica*, it is important to understand the difference in transcriptional regulation of woody SCW formation in hybrid parents and offspring. In this study, we performed comparative RNA-seq analysis of xylem, phloem, and leaf samples from M8 and 1601 plants at early latewood developmental stages to identify X-DEGs. Furthermore, X-SEGs were screened by comparing xylem expression data with those of the phloem and leaves to construct a gene co-expression network. Therefore, a comprehensive systems biology approach was used to fully characterize the variation in wood properties observed between M8 and 1601.

### 4.1 A complex regulatory network involving wood formation

Wood is formed by radial expansion within the cambium, which eventually leads to the development of the secondary xylem; this process involves the complicated synchronization of cell division, cell differentiation, programmed cell death, and SCW biosynthesis (Du et al., 2020). The complex and organized network of TFs governs all SCW development steps. Previous studies have identified several TFs involved in wood formation, including those involved in lignin biosynthesis, such as NAC and MYB (Zhang et al., 2014; Mizrachi and Myburg, 2016; Chen et al., 2019a).

The present study identified a total of 32 MYB genes (13 upregulated in 1601 and 19 upregulated in M8) and 20 NAC genes (five upregulated in 1601 and 15 upregulated in M8) (**Supplementary Table S2D**). Among these MYB genes, three were upregulated in M8: *MYB8* (F000339870), *MYB52* (F000365430), and *NAC29* (*XND1*, F000040830). *MYB8* regulates PAL expression, which is involved in phenylalanine metabolism and promotes wood formation. Alternatively, *MYB52* may be a repressor of wood formation, and recent studies have shown that *AtMYB52* is homologous to *PtrMYB161*. Overexpression of *PtrMYB161* reduces cambial activity (wood differentiation) and vessel diameter by enhancing lignin synthesis and inhibiting secondary growth (transverse thickening) in *Populus trichocarpa* (Wang et al., 2020). *MYB61* (F000040830) is upregulated in 1601 and activates CESA expression in rice spp. (Zhang et al., 2018a). Moreover, *XND1* has been shown to inhibit xylem vessel development when overexpressed (Zhao et al., 2008; Zhong et al., 2021), which may result in reduced vessel length and width and lower cellulose content in the M8 xylem. Intriguingly, *LHY* (F000262880), a circadian rhythm regulator, was observed at 1.12 times increased expression in 1601. In our previous study, *FmLHY* was determined to affect the growth resistance of hybrid *F. mandshurica* offspring under salt stress (He et al., 2016), which may have correspondingly prolonged the xylem growth cycle in 1601. WRKY TFs also play key roles in secondary wall synthesis, and WRKY TF mutants have been reported to be associated with increased lignin content (Wang et al., 2010). The differential expression of MYB, NAC, and WRKY TFs may explain the observed differences in wood properties between M8 and 1601. Ultimately, these results indicate that the mechanisms by which TFs mediate variations in the properties of hybrid wood are complex.

### 4.2 Variation in wood chemical compositions

SCWs, the main components of wood, are deposited through the action of various proteins involved in the synthesis, transport, and polymerization of cellulose, hemicellulose, and lignin (Zhong et al., 2011). The expression of protein-encoding genes is activated during the biosynthesis of cell wall components. Cellulose is mainly synthesized by the cellulose synthase complex within the Golgi apparatus and is then transported to the cell membrane. In the present study, we identified 37 X-DEGs associated with cellulose biosynthesis in *F. mandshurica* (M8) and *F. mandshurica* × *F. sogdiana* (1601) (**Supplementary Table S2E**). Most of the observed X-DEGs were annotated as CESA or CESA-like genes, of which three upregulated genes in the M8 xylem were identified as *CESA2*, *CESA3*, and *CSI3*, which are involved in cellulose synthesis in the primary cell wall (Xie et al., 2011). CSL-D3, E1, G2, and G3 were significantly upregulated in the 1601 xylem. Recent research has demonstrated that CSL genes, especially *CSL-D3*, are involved in the synthesis of cellulose and hemicellulose; in particular, *CSL-D3* plays a vital role in cellulose production and can fully rescue cellulose accumulation defects in *Arabidopsis* CESA mutants (Yang et al., 2020).

Lignin is primarily derived from hydroxycinnamyl alcohols via reactions involving the combination and coupling of radicals; this process is catalyzed by 10 enzyme families encoded by at least 21 monolignol genes within the phenylpropanoid biosynthesis pathway (Wang et al., 2018b). A total of 65 X-DEGs were classified as lignin biosynthesis-related genes (**Figure 5**; **Supplementary Table S2E**), among which 38 were upregulated in the xylem of *F. mandshurica* (M8). Two of these genes were identified as *PAL1* (F000026370) and *4CL2* (F000105140); however, *PAL1* was not specifically expressed in xylem tissue. Nonetheless, two other *PAL* genes (F000166720 and F000090920) were identified as X-SEGs. All five *PAL* genes are important for wood formation in *P. trichocarpa*; however, they have been determined to exhibit tissue-specific functions and expression patterns (Shi et al., 2013). Therefore, we speculate that the cooperation of the three *PAL* genes (F000026370, F000166720, and F000090920) may lead to the activation of the phenylpropane pathway, which could lead to an increase in lignin content in the M8 xylem. Additionally, *4CL2* was specifically expressed in the M8 xylem. In our previous study, we established that overexpression of *Fm4CL2* in tobacco increases lignin synthesis by increasing coniferyl alcohol content (Chen et al., 2020); therefore, we hypothesized that the upregulation of lignin synthesis-related genes, such as *PAL1* and *4CL2*, may increase lignin content in the M8 xylem. Of these 38 lignin biosynthesis-related DEGs, *CAD1* (F000295850) and *4CL4* (*Fm4CL4/4CL-like1*, F000291480) were significantly upregulated in the 1601 xylem. *CAD* primarily affects the composition of lignin monomers rather than the overall lignin content; alternatively, *4CL4* increases the number of xylem cells in *4CL4*-overexpression lines of tobacco (Pilate et al., 2002; Chen et al., 2019b). Studies on hybrid forest tree populations have shown a negative correlation between biomass growth (typically measured as wood volume) and lignin content, whereas cellulose is positively correlated with growth (Kirst et al., 2004; Novaes et al., 2010). Based on these findings, we speculate that the higher lignin content, resulting from more active phenylpropanoid synthesis, may lead to earlier lignification and a shorter growth cycle; ultimately, this may result in significantly lower growth rates in M8 trees than in 1601 trees. However, further research is required to determine whether *CAD1* and *4CL4* significantly contribute to lignin production in *F. mandshurica*.

### 4.3 WGCNA screening of key genes affecting wood development and properties

WGCNA of the xylem revealed significant differences between the core transcriptional networks of *F. mandshurica* (M8) and *F. mandshurica* × *F. sogdiana* (1601). In the wood formation-related module of M8 (blue), *CCR1*, *F5H*, *COMT2*, and *MYB52* were identified as hub genes (**Figures 6A, C**; **Supplementary Table S7A**), with *MYB52* being a hub TF that can either activate or inhibit the production of lignin and SCW, as determined by the target genes with which it interacts with (Wang et al., 2020). *CESA8* and COBRA-like protein 1 (*COBL1*) are hub genes in the wood formation-related module of 1601 (black) and contribute to cellulose synthesis (**Figures 6D, D**; **Supplementary Table S7B**). The function of *CesA8* cannot be overlooked, with *PrCesA8A/B* being a component of the SCW cellulose synthesis complex (Zhang et al., 2018b), which accounts for approximately half of the quantitative proteomic content readings in poplar observed via stoichiometry; this indicates that *CESA8* and *COBL1* initiate SCW synthesis and promote the biosynthesis of cellulose in 1601. Additionally, *WAT1*, *C3H14*, *C3H15*, and *MYB83* were identified as hub TFs in this 1601 SCW synthesis-promoting black module. Furthermore, the specific expression of *XCP2* in the M8 xylem suggests its potential contribution to the shorter growth cycle of M8 via the inhibition of xylem cell differentiation (Avci et al., 2008). Finally, *WRKY13* was determined to be located at a core gene position in the co-expression networks of both tree variants, indicating that this gene may play a significant role in lignin synthesis and secondary xylem development. Overall, these results suggest that the identified TFs may influence wood properties in both 1601 and M8.

It is interesting to note that some of the identified hub genes, such as *CESA8*, *C3H14*, and *C3H15*, alongside the X-DEGs *LHY* (1601), *MYB52* (M8), and *WAT1* (M8) are regulated by BRs (**Supplementary Table S7A, B**). BRs induce the expression of *CESA8*, which is involved in the secondary growth of *Arabidopsis* (Xie et al., 2011). Further, *C3H15* antagonizes BZR1 or BES1 during transcription and, thereby, represses cell elongation by modulating the BR signaling pathway (Chai et al., 2015; Chai et al., 2022). Similarly, *MYB52* has also been reported to be induced by BRs (Sun et al., 2010; Yu et al., 2011); further, *WAT1* is regulated by the crosstalk between BRs and auxin (Lee et al., 2021). Finally, *LHY* regulates circadian oscillations via the *BES1/TPL-CCA1/LHY* module, which controls plant growth in *Arabidopsis* (Lee et al., 2020). These findings collectively suggest that BRs play a crucial role in the wood development and properties of the hybrid parents and offspring of *F. mandshurica*.

### 4.4 Effect of BR synthesis and signaling on xylem development

Polyhydroxylated plant-derived steroid hormones, known as BRs, regulate both xylem growth and development (Jin et al., 2014; Shen et al., 2018; Jiang et al., 2021). Previous studies have linked the BR pathway to heterosis and natural domestication. For example, aberrant BR pathway activity in maize hybrids is a crucial contributor to the significant upregulation of productivity and biomass (Wang et al., 2018a). BR synthesis enzyme genes *DWF4*, *CPD*, and *DET2*; and a BR receptor, BRI homologous gene *BRL1.2*, has been found to promote SCW formation and xylem growth in poplar (Si et al., 2016; Shen et al., 2018; Du et al., 2020; Jiang et al., 2021). In the present study, 27 X-DEGs were found to be involved in the BR pathway (**Supplementary Table S2F**). Three of the identified X-DEGs, *CPD* (F000110180 and F000362610) and *BRI1* (F000324690), were upregulated in *F. mandshurica* × *F. sogdiana* (1601). In addition, the X-SEGs observed in *F. mandshurica* (M8) and *F. mandshurica* × *F. sogdiana* (1601) exhibited clear gene overlap (**Supplementary Table S5A, B**), including that of *BEH4* (F000335340), *BES1* (F000356920), *SPL2* (F000075150), *SPL16* (F000343240), and *BRI1* (F000324690); however, certain discrepancies were evident, such as *DET2* (F000227880) being specifically expressed only in the xylem of 1601. In addition, the GSK3 kinase BIN2/AtSK21 phosphorylates cellulose synthase, which inhibits cellulose production in *Arabidopsis* (Sánchez-Rodríguez et al., 2017). In the present study, multiple *GSK3* kinase family homologous genes were determined to be highly expressed in the M8 variant; this may have contributed to the low cellulose content in the M8 xylem. However, the upregulation of *DWF4* in M8 cells appears to contradict its established role; an earlier study demonstrated that overexpression of *PtoDWF4* elevated BR levels *in vivo*, which increased both lignin and cellulose content in *Populus tomentosa* (Shen et al., 2018). In contrast, the exogenous application of BL significantly reduces the total lignin content of *Liriodendron tulipifera* (Jin et al., 2014). These data indicate a difference between the increase in endogenous BR levels and the application of exogenous BRs in plants; alternatively, there may be species-specific regulatory mechanisms of BRs that differ between tree species. Therefore, an in-depth investigation is necessary to determine if GSK3 kinase is involved in regulating biological processes associated with the wood formation in *F. mandshurica*.

Previous studies have demonstrated that BRs promote a reduction in plant lignification (Jin et al., 2014). Considering the differences in xylem development, we measured the BR content in the xylem of M8 and 1601 from May to August. The results showed that 1601 exhibited a higher BR content than M8 at all four time points (**Figure 8E**), which may explain why the lignification process of 1601 occurred later than that of M8. *CPD* (also known as *CYP90A1*) encodes a C-23 hydroxylase, which plays an important role in the hydroxylation of various sterols (Szekeres et al., 1996); *CPD* genes are understood to encode the rate-limited reaction enzymes of these pathways (Shi et al., 2015). In a previous study, transgenic plants overexpressing these *CPD* genes exhibited a significant increase in vegetative growth, inflorescence stem height, and biomass yield owing to an increase in endogenous levels of BR biosynthesis (Si et al., 2016). In the present study, another *CPD* gene, *FmCPD* (F000362610), was cloned from *F. mandshurica*. Interestingly, we observed that *FmCPD* expression reached its peak after 48 h of transient cultivation, whereas BR content reached its peak within 24 h (**Figures 8F, G**). Ultimately, we hypothesized that the transcription of *FmCPD* is feedback regulated by BRs (Bancoş et al., 2002). The qRT-PCR results from transient *FmCPD* overexpression revealed significant upregulation of *FmCESA4*, *FmCESA7*, *FmCESA8*, *FmCOMT1*, *FmIRX9*, *FmXTH4*, and *FmMYB20* among the 12 SCW synthase genes. Slight upregulation was also observed for *FmCAD*, *Fm4CL1*, *Fm4CL2*, *FmCCR1*, and *FmMYB52* in transgenic OE-*FmCPD* lines (**Figure 8H**). *CESA4*, *CESA7*, and *CESA8* are core components of the SCW cellulose synthase complex, which has been identified in poplar and *Arabidopsis* (Olins et al., 2018; Nayeri et al., 2022). In the present study, *CAD*, *4CL1*, *4CL2*, and *CCR1*, which control the synthesis of lignin monomers, were slightly upregulated in 1601; this is consistent with the high cellulose and low lignin contents observed in 1601. Notably, *FmMYB20* expression was also induced by BRs.

Extensive evidence has shown that the exogenous application of BL induces growth in *Liriodendron* stems and influences xylem formation during secondary growth in poplar species (Jin et al., 2014; Du et al., 2020). The results of the exogenous application of BRs in *F. mandshurica* also support this understanding. In this study, we established that BRs affect xylem development and wood variation in *F. mandshurica*. After applying 10 μM of BL for 3 weeks, the growth of *F. mandshurica* seedlings significantly accelerated (**Figures 9A, B**); additionally, during this treatment, cellulose and hemicellulose contents increased, and lignin content decreased in the xylem (**Figures 9E–G**). Histochemical staining with fast green/safranin indicated an increase in the number of xylem cell layers in plants treated with BL compared with mock control plants (**Figures 9C, D, H, I**). Conversely, the mock control plants exhibited a reduced xylem area (**Figure 9H**) and fewer xylem cell layers (**Figure 9I**). Differences in BR synthesis may have contributed to this variation in xylem traits by altering downstream signal transduction pathways. Previous research has demonstrated that BRs affect carbohydrate biosynthesis in the secondary xylem cell wall (Jin et al., 2014); our findings similarly revealed differences in the starch and sucrose metabolic pathways in the xylem of M8 and 1601 (**Supplementary Table S2G**), which may also be caused by BR signal transduction.

Taken together, these data suggest that BR synthesis positively regulates xylem development during wood formation. Enhanced BR synthesis led to variations in wood formation between *F. mandshurica* parents and their hybrid offspring. Furthermore, increased BR content affected BR signaling. Specifically, *BRI1* may be an important factor in this signaling pathway that affects *BZR1*/*BES1* and other downstream genes (e.g., X-DEGs and X-SEGs) that are involved in signal transduction. Nonetheless, further investigations are necessary to thoroughly understand the precise regulation of xylem development by BR synthesis and signaling in woody hybrid plants.

### 4.5 Conclusion

Through interspecific hybridization of *F. mandshurica*, a novel variant, *F. mandshurica* × *F. sogdiana* (1601), was obtained. By evaluating the dynamic variations in wood and RNA-seq of three tissue types (phloem, leaf, and xylem), we analyzed the wood formation process associated with transcriptional programs in the latewood development stage. Compared with *F. mandshurica* (M8), *F. mandshurica* × *F. sogdiana* (1601) exhibited a reduced lignin content, increased cellulose content, and an extended growth cycle (by 2 weeks), which appeared to be related to its higher endogenous BR content. We used the BR synthesis enzyme gene *FmCPD* to transiently transform *F. mandshurica*; this demonstrated that BRs regulate the genes involved in wood development. Exogenously applied BL also significantly accelerated the growth of *F. mandshurica* seedlings and altered wood properties in *F. mandshurica.* Overall, our results demonstrate that BRs are essential components that affect wood development and properties in interspecific hybrid *F. mandshurica* (1601) and its female parent *F. mandshurica* (M8).

## Data availability statement

The datasets presented in this study can be found in online repositories. The names of the repository/repositories and accession number(s) can be accessed through the following link: https://www.ncbi.nlm.nih.gov/bioproject/PRJNA815743.

## Author contributions

HL and MC designed the experiments. HL and FZ conducted the experiments. HL and JY analyzed the transcriptome data. HL drafted the manuscript. MF, WS, and MC maintained the plant materials. HL and FZ discussed the results and finalized the manuscript. All authors contributed to the article and approved the submitted version.
