# Supplementary material for: Brassinosteroids affect wood development and properties of Fraxinus mandshurica
Source: Front Plant Sci. 2023 Jul 17;14:1167548. doi: 10.3389/fpls.2023.1167548 (PMC10400452; doi:10.3389/fpls.2023.1167548)
Supplement: Supplementary file 1 [file DataSheet_1.docx]

# Supplementary Figures and Tables

## Supplementary Figures


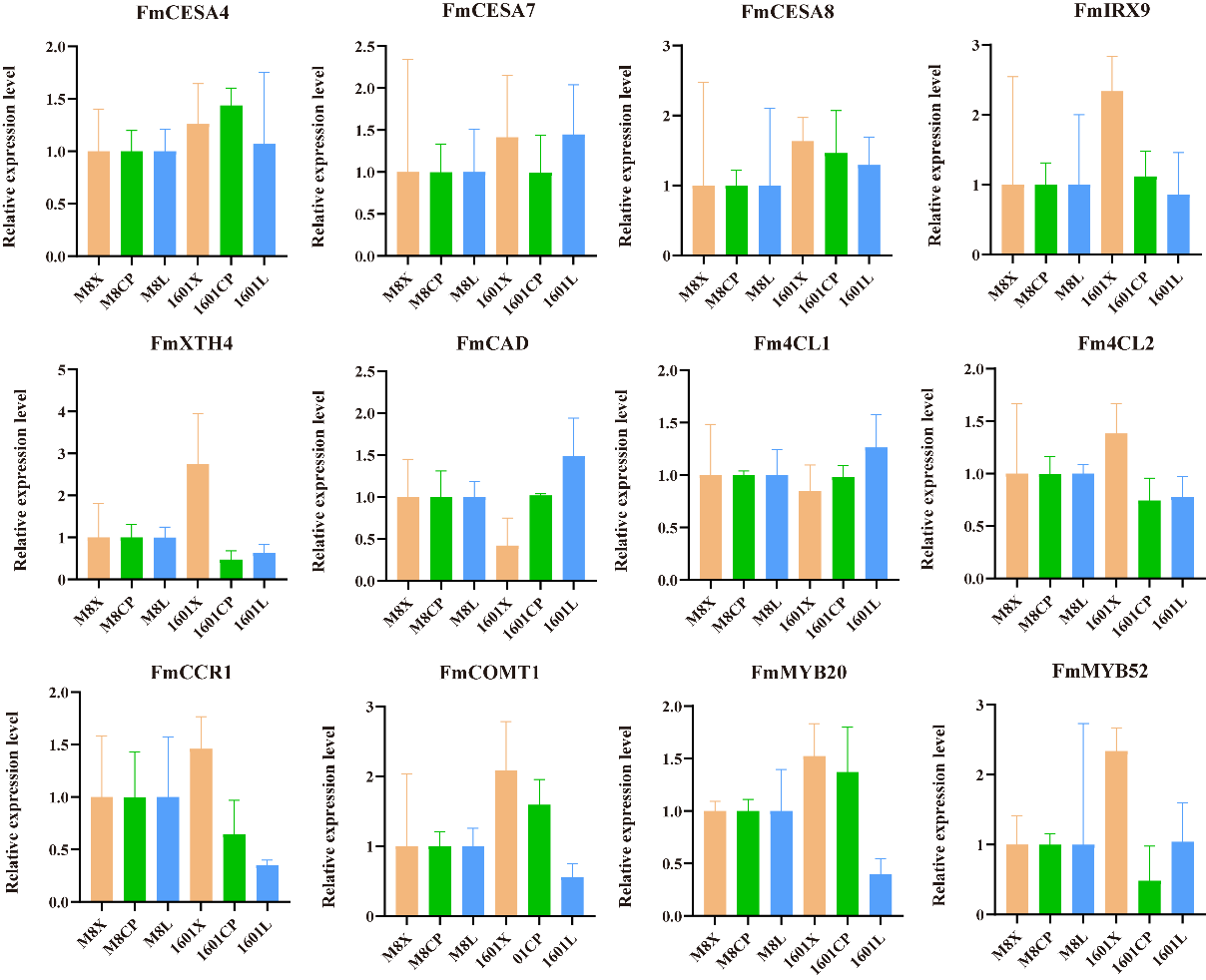


**Supplementary Figure 1**. qRT-PCR for selected genes. The results are consistent with transcriptome sequencing results, indicating that transcriptome sequencing results are highly reliable.


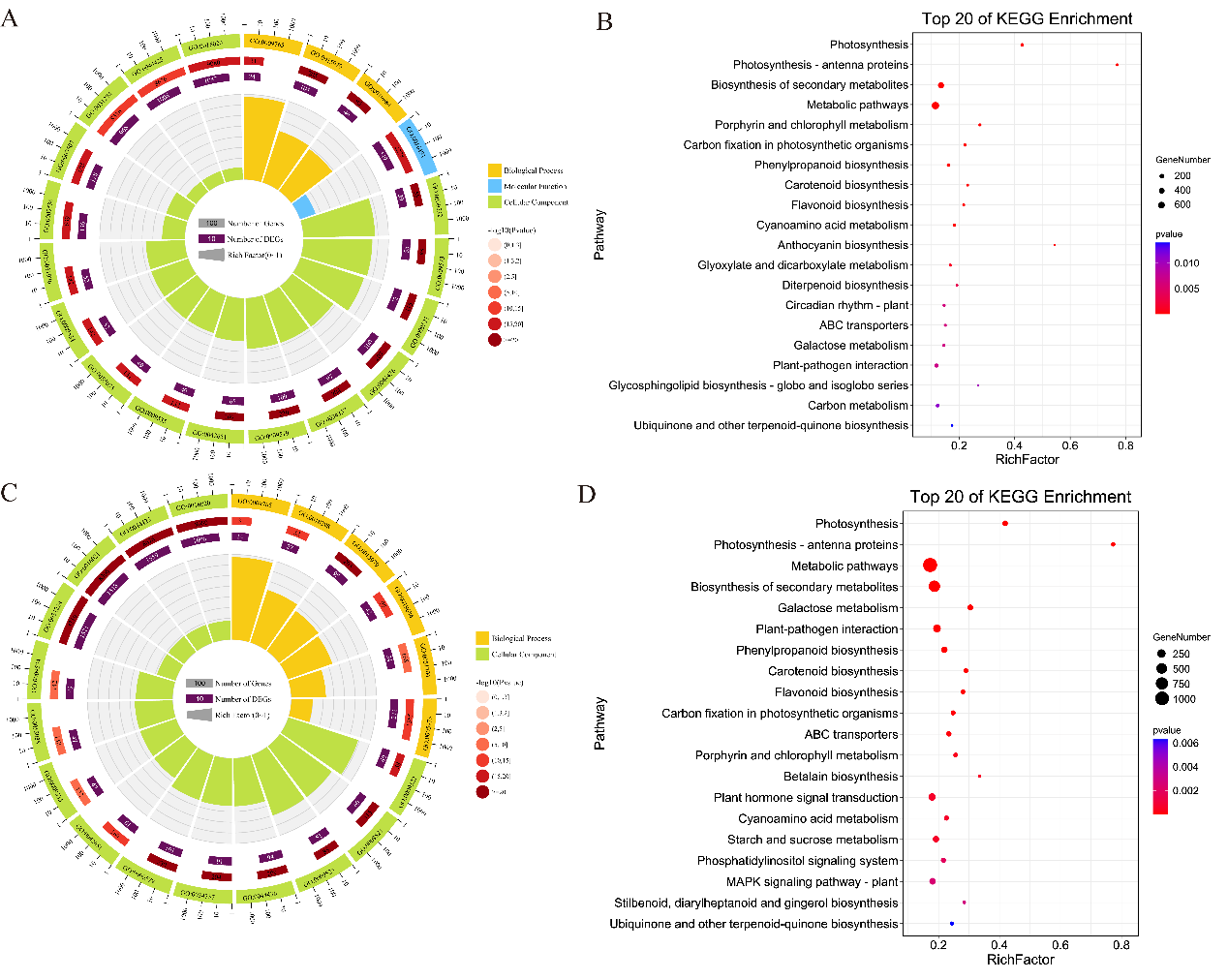


**Supplementary Figure 2**. GO and KEGG analysis of Xylem-Specifically Expressed Genes (X-SEGs) for *F. mandshurica* (M8) and *F. mandshurica × F. sogdiana* (1601). A and C display a more detailed classification of GO analysis for *F. mandshurica* (M8) and *F. mandshurica × F. sogdiana* (1601) respectively. B and D showed KEGG results for *F. mandshurica* (M8) and *F. mandshurica × F. sogdiana* (1601) respectively. Topological structure of top 3 highest enrichment factor GO terms (molecular function, cellular component and biological process) were shown in A and C. Top20 pathways were shown in the graph B and D.


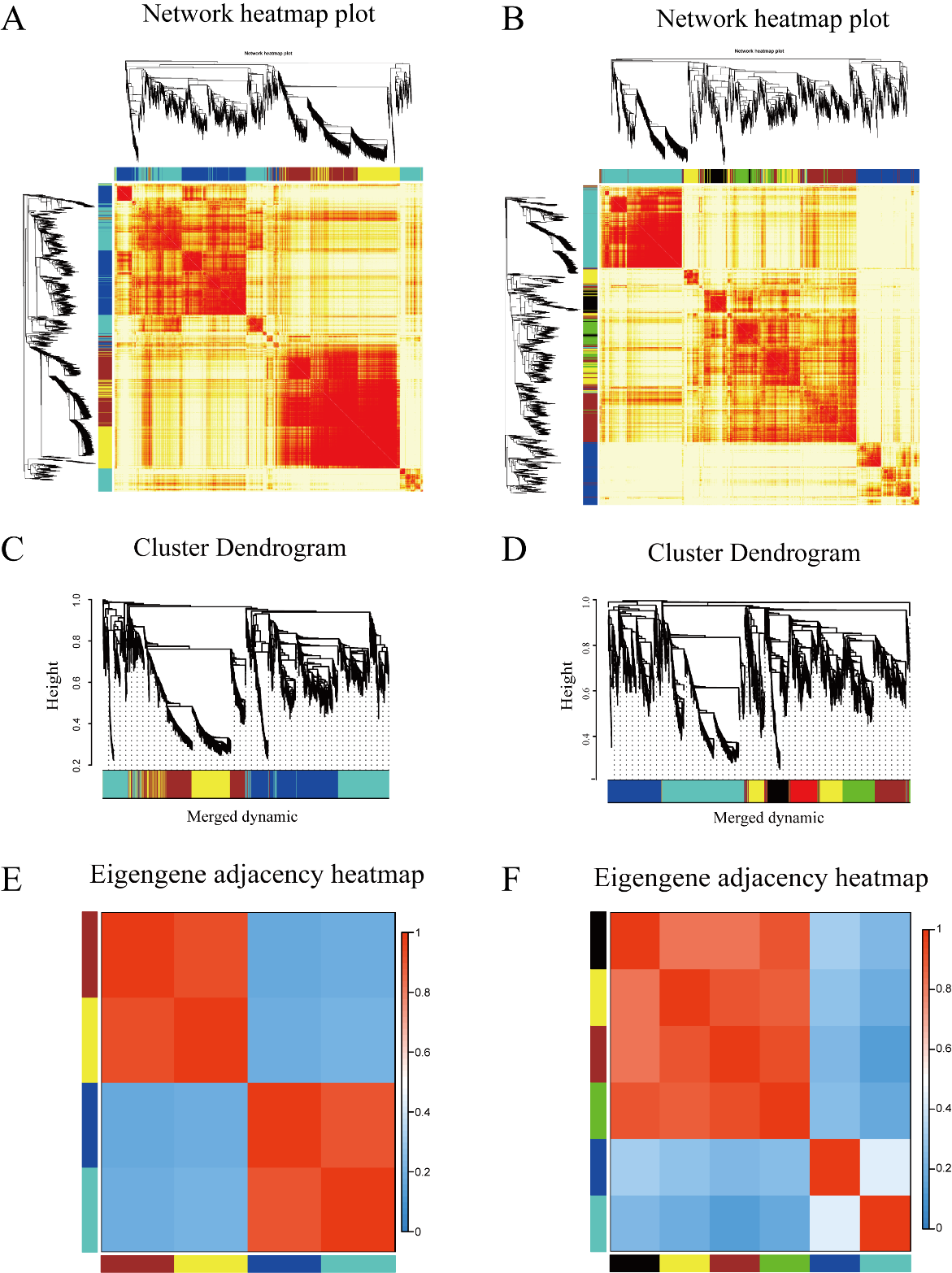


**Supplementary Figure 3.** Result of WGCNA analysis for *F. mandshurica* (M8) and *F. mandshurica × F. sogdiana* (1601). A, C and E are WGCNA analysis results of *F. mandshurica* (M8). B, D and F are WGCNA analysis results of *F. mandshurica × F. sogdiana* (1601). A and B are heatmaps of gene co-expression network. The heatmaps represent adjacencies among genes in analysis: one single gene corresponds to other genes in each row and column. Dark color means high correlation between two genes. Light-luminance color means low correlation between two genes. The different colors in C and D represent different modules. E and F are correlation analyses result of different module for *F. mandshurica* (M8) and *F. mandshurica × F. sogdiana* (1601). The red color means high correlation between two modules. The blue color means low correlation between two modules.


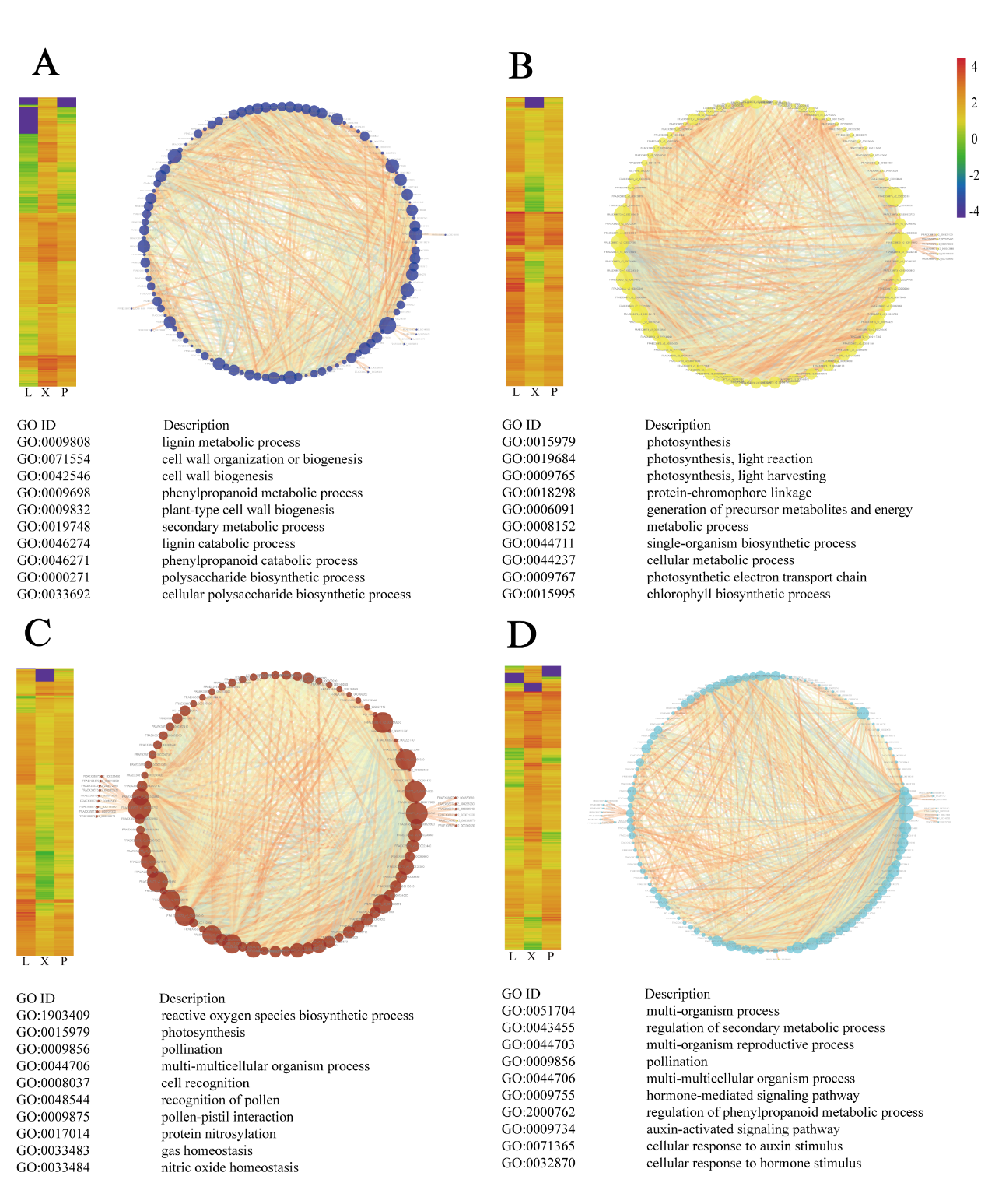


**Supplementary Figure 4.** Xylem-Specifically Expressed Genes (X-SEGs) Co-expression network and GO analysis in *F. mandshurica* (M8). (A-D) Co-expression network in F. mandshurica (M8). In this network, only the top 1,000 co-expression gene nodes with KME value are displayed. Only the top ten GO terms under the biological process category appear in each module. The heatmaps are for gene expression levels in the different modules.


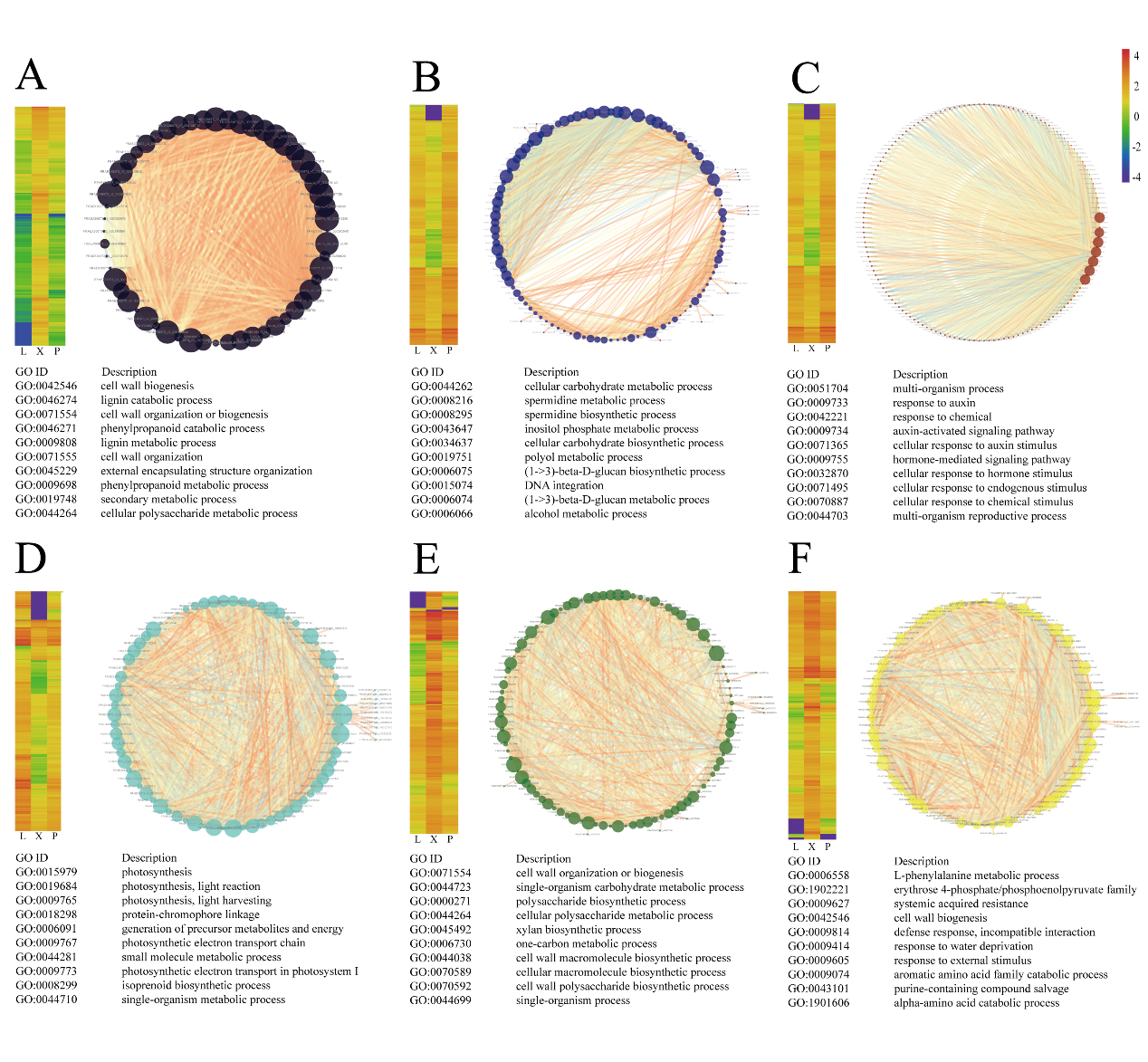


**Supplementary Figure 5.** Xylem-Specifically Expressed Genes (X-SEGs) Co-expression network and GO analysis in *F. mandshurica* × *F. sogdiana* (1601). (A-D) Co-expression network in *F. mandshurica* × *F. sogdiana* (1601). In this network, only the top 1,000 co-expression gene nodes with *_kme_* value are displayed. Only the top ten GO terms under the biological process category appear in each module. The heatmaps are for gene expression levels in the different modules.

- 1. **Supplementary Tables**

**Supplementary Table S1**. Summary information of the RNA-Seq results for the *F. mandshurica* (M8) and *F. mandshurica × F. sogdiana* (1601).

**Supplementary Table S2.** Xylem Differentially Expressed Gene analysis Between *F. mandshurica* (M8) and *F. mandshurica × F. sogdiana* (1601). **Supplementary Table S2A.** Differentially Expressed Gene analysis result in xylem groups (Xylem-Differentially Expressed Genes (X-DEGs) analysis result) of *F. mandshurica* (M8) and *F. mandshurica* × *F. sogdiana* (1601). **Supplementary Table S2B.** GO enrichment analysis of X-DEGs. **Supplementary Table S2C.** KEGG Out Pathway Enrichment of X-DEGs. **Supplementary Table S2D.** Transcription factors enriched to X-DEGs between *F. mandshurica* (M8) and *F. mandshurica × F. sogdiana* (1601). **Supplementary Table S2E.** Lignin, Cellulose, and Hemi-Cellulose Synthase Pathway analysis in X-DEGs. **Supplementary Table S2F.** Hormone Synthesis and Signaling Pathway analysis in X-DEGs. **Supplementary Table S2G.** Starch and Sucrose metabolism Pathway analysis in X-DEGs.

**Supplementary Table S3.** Xylem-Specifically Expressed Genes (X-SEGs) analysis result for *F. mandshurica* (M8) and *F. mandshurica × F. sogdiana* (1601), respectively**. Supplementary Table S3A.** Differentially Expressed Gene analysis result in L vs X group of *F. mandshurica* (M8). **Supplementary Table S3B.** Differentially Expressed Gene analysis result in CP vs X group of *F. mandshurica* (M8) **Supplementary Table S3C.** Differentially Expressed Gene analysis result in L vs. X group of *F. mandshurica × F. sogdiana* (1601) **Supplementary Table S3D.** Differentially Expressed Gene analysis result in CP vs X group of *F. mandshurica × F. sogdiana* (1601).

**Supplementary Table S4.** GO and KEGG analysis results of X-SEGs. For *F. mandshurica* (M8) and *F. mandshurica × F. sogdiana* (1601). **Supplementary Table S4A.** GO analysis for up and down regulated genes in xylem of *F. mandshurica* (M8). **Supplementary Table S4B.** KEGG analysis for up and down regulated genes in xylem of *F. mandshurica* (M8). **Supplementary Table S4C.** GO analysis for up and down regulated genes in xylem of *F. mandshurica × F. sogdiana* (1601). **Supplementary Table S4D.** KEGG analysis for up and down regulated genes in xylem of *F. mandshurica × F. sogdiana* (1601).

**Supplementary Table S5.** X-SEGs distribution in wood-formation related pathway in *F. mandshurica* (M8) and *F.mandshurica × F. sogdiana* (1601)**. Supplementary Table S5A.** X-SEGs distibution in wood formation related pathway in *F. mandshurica* (M8). **Supplementary Table S5B.** X-SEGs distibution in wood formation related pathway in *F.mandshurica* × *F. sogdiana* (1601).

**Supplementary Table S6.** GO and KEGG analysis of WGCNA in different modules of *F. mandshurica* (M8) and *F. mandshurica × F. sogdiana* (1601). **Supplementary Table S6A.** GO analysis result in blue module of *F. mandshurica* (M8). **Supplementary Table S6B.** KEGG analysis result in blue module of *F. mandshurica* (M8). **Supplementary Table S6C.** GO analysis result in brown module of *F. mandshurica* (M8). **Supplementary Table S6D.** KEGG analysis result in brown module of *F. mandshurica* (M8). **Supplementary Table S6E.** GO analysis result in turquoise module of *F. mandshurica* (M8). **Supplementary Table S6F.** KEGG analysis result in turquoise module of *F. mandshurica* (M8). **Supplementary Table S6G.** GO analysis result in yellow module of *F. mandshurica* (M8). **Supplementary Table S6H.** KEGG analysis result in yellow module of *F. mandshurica* (M8). **Supplementary Table S6I.** GO analysis result in black module of *F. mandshurica × F. sogdiana* (1601). **Supplementary Table S6J.** KEGG analysis result in black module of *F. mandshurica × F. sogdiana* (1601). **Supplementary Table S6K.** GO analysis result in brown module of *F. mandshurica × F. sogdiana* (1601). **Supplementary Table S6L.** KEGG analysis *result in brown module of F. mandshurica × F. sogdiana (1601).* ***Supplementary* Table S6M.** GO analysis result in green module of *F. mandshurica × F. sogdiana* (1601). **Supplementary Table S6N.** KEGG analysis result in green module of *F. mandshurica × F. sogdiana* (1601). **Supplementary Table S6O.** GO analysis result in blue module of *F. mandshurica × F. sogdiana* (1601). **Supplementary Table S6P.** KEGG analysis result in blue module of *F. mandshurica × F. sogdiana* (1601). **Supplementary Table S6Q.** GO analysis result in turquoise module of *F. mandshurica × F. sogdiana* (1601). **Supplementary Table S6R.** KEGG analysis result in turquoise module of *F. mandshurica × F. sogdiana* (1601). **Supplementary Table S6S.** GO analysis result in yellow module of *F. mandshurica × F. sogdiana* (1601). **Supplementary Table S6T.** KEGG analysis result in yellow module of *F. mandshurica × F. sogdiana* (1601).

**Supplementary Table S7.** wood-formation related genes co-expression network of *F. mandshurica* (M8) and *F. mandshurica × F. sogdiana* (1601), respectively. **Supplementary Table S7A.** Specifically expressed genes in co-expression network of *F. mandshurica* (M8) Blue Module. **Supplementary Table S7B.** Specifically expressed genes in co-expression network of *F.mandshurica × F. sogdiana* (1601) Black Module.

**Supplementary Table S8**. Primer sequences were used in this study. S**upplementary Table S8A**. Primers for validation of RNA-seq; **Supplementary Table S8B**. Primers sequences used of overexpressing in FmCPD (OE-*FmCPD*) and CON (Control).
